# Projecting future damage costs of non‐native species using combined dynamical and cost–density equations

**DOI:** 10.1002/eap.70252

**Published:** 2026-07-06

**Authors:** Danish A. Ahmed, Corey J. A. Bradshaw, Noor Tahat, Emma J. Hudgins, Pierre Courtois, Philip E. Hulme, Yuya Watari, Ali Serhan Tarkan, Ismael Soto, Phillip J. Haubrock, Paride Balzani, Ross N. Cuthbert

**Affiliations:** ^1^ Department of Mathematics and Natural Sciences Gulf University for Science and Technology Hawally Kuwait; ^2^ Global Ecology Partuyarta Ngadluku Wardli Kuu, College of Science and Engineering Flinders University Adelaide South Australia Australia; ^3^ Australian Research Council Centre of Excellence for Indigenous and Environmental Histories and Futures Cairns Queensland Australia; ^4^ School of Agriculture, Food, and Ecosystem Sciences The University of Melbourne Parkville Victoria Australia; ^5^ Centre for Environmental Economics Montpellier University, CNRS, INRAE Montpellier France; ^6^ The Centre for One Biosecurity Research, Analysis and Synthesis, Department of Pest‐Management and Conservation Lincoln University Canterbury New Zealand; ^7^ Forestry and Forest Products Research Institute Tsukuba Japan; ^8^ Faculty of Biology and Environmental Protection, Department of Ecology and Vertebrate Zoology University of Lodz Lodz Poland; ^9^ Department of Basic Sciences, Faculty of Fisheries Muğla Sıtkı Koçman University Muğla Turkey; ^10^ Faculty of Fisheries and Protection of Waters, South Bohemian Research Center of Aquaculture and Biodiversity of Hydrocenoses University of South Bohemia in České Budějovice Vodňany Czech Republic; ^11^ Department of Conservation Biology and Global Change Doñana Biological Station (CSIC) Seville Spain; ^12^ Department of Life and Environmental Sciences, Faculty of Science and Technology Bournemouth University Poole Dorset UK; ^13^ Institute for Global Food Security, School of Biological Sciences Queen's University Belfast Belfast UK

**Keywords:** biological invasions, damage modeling, economic costs, impact projections, *InvaCost*, invasion management, logistic growth, non‐native species

## Abstract

Biological invasions threaten biodiversity, economic stability, and public health, exacerbated by intensive global trade and transport. The economic costs of these invasions have exceeded US$2 trillion globally and continue to increase. Although past invasion costs have been described across various contexts, there are few robust projections of future costs, limiting effective management planning. We developed a mathematical framework to project future economic damage caused by biological invasions, combining cost–density relationships with a density–time function based on logistic population growth. We tested the model on five well‐documented non‐native mammal species in Japan, a country with long‐term, high‐resolution invasion cost records and a well‐characterized history of mammal introductions: Pallas' squirrel *Callosciurus erythraeus*, small Indian mongoose *Herpestes javanicus*, nutria *Myocastor coypus*, masked palm civet *Paguma larvata*, and raccoon *Procyon lotor*. Species‐level cost–density relationships were characterized by two distinct forms: a high‐density curve for *M. coypus* and *P. lotor*, where costs increase progressively with density but the rate of escalation slows at higher densities, and a high‐threshold curve for *C. erythraeus*, *H. javanicus*, and *P. larvata*, where costs remain minimal until populations exceed a density threshold, after which they rise steeply. Our model projected accumulated costs to 2050 varying over several orders of magnitude, from US$0.43 million (*H. javanicus*) to US$88 million (*P. larvata*), with proportional increases ranging from ~15% (*M. coypus*) to ~78% (*H. javanicus*). Under business‐as‐usual management, we explicitly model damage‐only costs, assuming a historically observed management trend. These projections should therefore be interpreted as maximum estimates. Our approach identifies thresholds beyond which damages escalate rapidly—costs begin to surge 40–80 years after the first record, with 90% of expected long‐term damages incurred typically within 10–20 years. For managers, these results highlight the importance of timely interventions, underscoring the need for tailored management strategies considering species‐specific dynamics, socioeconomic contexts, and the speed of cost escalation. Early‐stage cost dynamics can project future trajectories of existing and emerging invasions, helping guide proactive management prioritization. Our projections equip policymakers and resource managers with improved foresight to anticipate and mitigate future economic burdens of non‐native species across spatial scales and for different taxa.

## 要旨

外来種の侵入は，生物多様性や経済の安定性，公衆衛生の脅威となり，活発化する国際貿易と輸送によって悪化している.このような，外来種の侵入によりもたらされる経済被害は，世界全体で数兆米ドルに達しており，今後も増加し続けると予想されている.しかし，すでに生じた過去の外来種の侵入による経済被害については，これまでにさまざまな文脈で説明されてきたものの，将来生じうる経済被害に関する信頼性の高い推計はほとんどなく，効果的な管理計画の立案を困難にしていた.本研究では，経済被害と密度の関係と，ロジスティック個体群成長に基づく密度‐時間関数とを組み合わせることで，外来種の侵入による将来の経済被害を推計する数理的枠組みを開発した.私たちは外来種の侵入に伴う経済コストに関する長期的かつ高解像度の記録が整備され，哺乳類の導入の歴史もよく解明されている日本の外来哺乳類を対象に，その中で記録の充実している5種の外来哺乳類―クリハラリス *Callosciurus erythraeus*，フイリマングース *Herpestes javanicus*，ヌートリア *Myocastor coypus*，ハクビシン *Paguma larvata*，アライグマ *Procyon lotor*―を用いて，このモデルの検証を行った.種ごとの被害額と密度の関係は，大きくふたつの異なるタイプに分類された.ひとつは，密度の増加に伴って被害額が段階的に上昇するものの，高密度域ではその上昇率が緩やかになる「高密度型カーブ」で，ヌートリアとアライグマが該当した.もう一つは，密度が一定の閾値を超えるまでは被害がほとんど発生せず，閾値を超えると急激に上昇する「高閾値型カーブ」で，タイワンリス，フイリマングース，ハクビシンが該当した.本モデルによる2050年までの累積経済被害の予測値は，数桁規模で変動し，最小で43万ドル(フイリマングース)，最大で8800万ドル(ハクビシン)となり，コストの増加率は約15%(ヌートリア)から約78%(マングース)の範囲であった.通常の外来種管理を前提とした本モデルでは，過去に観察された管理の傾向をもとに，外来種の被害に関する被害額のみを明示的にモデル化している.したがって，これらの推計は，最大値として解釈する必要がある.本研究の手法によって，被害が急激に拡大する閾値が明らかになり，最初の外来種の野外での記録から40〜80年後に被害額が急激に増加しはじめ，通常その後10〜20年の間に，想定される長期的被害額の90%が生じることが示された.本研究の結果は，外来種対策の管理者にとって，タイミングを逸しない早期の対応が重要であることを示しており，種ごとの動態や社会経済的な背景，および経済被害の増加スピードを考慮した，きめ細やかな管理戦略の必要性を強調している.侵入初期段階における経済被害額の動態を把握することで，すでに生じている侵入および新たに生じる侵入の将来推移を推計することができ，先手を打った管理の優先順位づけに役立てることができる.本研究の推計は，政策立案者および資源管理担当者に対し，空間スケールや分類群を問わず，外来種による将来的な経済的損害を事前に見積もり，その軽減に備えるための先見性をもたらすものである.

## INTRODUCTION

The global expansion of non‐native species is driving biodiversity loss worldwide, having contributed to at least 60% of recorded extinctions (Bellard et al., 2016, 2022; Simberloff et al., 2013). The introduction rate of non‐native species is also increasing, with 200 species introduced to areas outside their native range annually (IPBES, 2022), driven mainly by trade and transportation networks that link previously isolated biological communities (Seebens et al., 2017, 2018, 2020). Biological invasions generally follow four main stages: transport, introduction, establishment, and spread; however, a non‐native species' impacts can appear as soon as the species is introduced to a novel environment, as they interact with native species, modify environments, and/or exploit available resources (Blackburn et al., 2011; Soto, Balzani, et al., 2024).

There are 13 main biodiversity‐impact mechanisms identified for non‐native species, including direct effects such as competition, predation, or disease transmission, and passive impacts such as toxicity (when consumed) or induced biofouling (Blackburn et al., 2014). These impacts operate across multiple ecological scales and provide the underlying mechanisms through which non‐native species generate broader societal and economic consequences (Blackburn et al., 2014; Carneiro et al., 2025). For instance, non‐native species can adversely affect native populations by reducing their population sizes or causing local extinctions (Bellard et al., 2016; Vanbergen et al., 2017). By altering biotic and abiotic interactions, such ecological disruptions can initiate cascading effects that compromise ecosystem structure and function and might amplify future invasions, thereby increasing long‐term economic damages (Bucciarelli et al., 2019; Stigall, 2010; Vanbergen et al., 2017; Walsh et al., 2016). Furthermore, non‐native species can negatively affect human health by acting as vectors or reservoirs for various pathogens and parasites (Roy et al., 2023), causing direct physical harm through venoms or toxins, or by exacerbating allergies (Juliano & Philip Lounibos, 2005; Roy et al., 2023; Schaffner et al., 2020).

In addition to the severe ecological and health repercussions of non‐native species, they are also responsible for a growing economic burden across economic sectors, such as agriculture, forestry, and fisheries (Ahmed et al., 2023; Bradshaw et al., 2016; Diagne et al., 2021; Turbelin et al., 2024). Monetary cost assessments of biological invasions help to promote more sustainable global management actions that result in better environmental and health outcomes (Soto, Haubrock, et al., 2023), even though many countries struggle to prevent and/or manage invasions (Bradshaw et al., 2024; Early et al., 2016). Although most countries have set targets to manage biological invasions, 45% of countries do not invest at all in the management of non‐native species (IPBES, 2022). There is also a lack of international coordination in management, with most countries acting independently and with predominantly reactionary approaches (Cuthbert, Diagne, Hudgins, et al., 2022). This historical neglect of non‐native species management erroneously stems from a perception that the costs of intervention outweigh the potential benefits, in part owing to a lack of damage‐cost synthesis and insufficient projections (Carneiro et al., 2024; Heikkilä, 2011). However, early investment in management strategies, such as biosecurity, is often more cost‐effective than long‐term control measures (Ahmed, Hudgins, Cuthbert, Kourantidou, et al., 2022; Bradshaw et al., 2024; Cuthbert, Diagne, Hudgins, et al., 2022; Leung et al., 2002). Most invasion‐cost assessments have so far been descriptive (Ahmed et al., 2023), and there is accordingly a need to move towards projective analyses of costs through space and time with accessible models. With rapidly escalating impacts, identifying the future temporal dynamics of costs could inform the necessary timings for management interventions across invasion stages to avoid severe impacts, while enabling the projection of future cost trajectories for existing and emerging invasions.

A major challenge to management is that the impacts of non‐native species are dynamic as populations shift in abundance or range (Dickey et al., 2020; Parker et al., 1999; Soto, Macêdo, et al., 2024). In some cases, these populations exhibit boom–bust dynamics, where an initial rapid increase in population size is followed by a decline or stabilization due to resource depletion, natural enemies, or other ecological factors (Copp, 2007; Strayer et al., 2017). These fluctuations further complicate projections of long‐term impacts and the effectiveness of control measures. The dynamics are often nonlinear and influenced by delays in detecting the presence of non‐native species or their impacts that hinder the implementation of control measures (Soto, Ahmed, et al., 2023). Indeed, negative impacts can be initially slow to accrue owing to time lags in demographic processes as the new species adapt to novel environmental conditions before expanding rapidly (Haubrock et al., 2022; Robeck et al., 2024), leading to a “cost of inaction” from delayed management (Ahmed, Hudgins, Cuthbert, Kourantidou, et al., 2022). These invasion relationships with time can be fundamentally approximated using a logistic curve, characterized by an initial exponential population growth followed by eventual stabilization (Soto, Ahmed, et al., 2023). However, while invasion dynamics have previously been characterized in terms of establishment and spread (e.g., the sigmoidal “invasion curve”; Haubrock et al., 2022), the interplay between economic damage and the population dynamics of non‐native species under changing environmental and economic conditions is still poorly understood. Indeed, broad‐scale studies on invasion costs frequently present only aggregated and static monetary estimates, while neglecting the temporal dynamics of damage costs and their relationship to population growth (Ahmed, Hudgins, Cuthbert, Haubrock, et al., 2022). Although more recent cost assessments explicitly consider temporal dynamics, few studies have projected the economic impacts of biological invasions (Henry et al., 2023; Tarkan et al., 2024), particularly while accounting for the invasion dynamics of the species involved. This often leads to broad generalizations that are not always useful to managers because such projections ignore variation in cost dynamics among taxonomic groups and contexts.

Mammals are among the most well‐studied taxonomic groups in invasion science, owing to their broad ecological impacts across various mechanisms, well‐documented global distributions and conspicuousness, and diverse introduction pathways with longstanding invasion histories (Biancolini et al., 2024; Blackburn et al., 2017; Tedeschi et al., 2022; Wang et al., 2023). Globally, mammals are the costliest class of invasive vertebrates (Diagne et al., 2021; Soto et al., 2025), with their introduction rates peaking substantially earlier than other taxa owing to widespread intentional introductions (Seebens et al., 2017). This ensures that the temporal granularity of economic impact data is high, even at the national scale where management decisions are typically implemented (Ahmed, Hudgins, Cuthbert, Haubrock, et al., 2022). Here, we developed and tested a cost model to project future impacts from five non‐native mammals in Japan for which we have extensive data on damage costs: Pallas' squirrel *Callosciurus erythraeus*, small Indian mongoose *Herpestes javanicus*, nutria *Myocastor coypus*, masked palm civet *Paguma larvata*, and raccoon *Procyon lotor*. These species all cause severe monetary and ecological costs by outcompeting native species, preying on wildlife, overgrazing, and spreading diseases (Doi et al., 2024; Katahira et al., 2022; Nagayama et al., 2020; Tatemoto et al., 2022; Watari et al., 2021). We focus on Japan given the high‐resolution annual data availability for these taxa within the *InvaCost* database (Diagne et al., 2020), strong national interest in biological invasions and their management (Watari et al., 2021), and its island status that spatially constrains population expansion as well as limits diffusion from neighboring countries. Beyond this context, our approach can be readily extended to other taxa and regions.

To promote timely actions that reduce future impacts, it is necessary to develop robust impact projections. Ahmed, Hudgins, Cuthbert, Haubrock, et al. (2022) previously developed a modeling framework linking invasion costs to population dynamics. Here, we advance this framework to project future costs at the species level by developing a model that integrates cost–density relationships with a density–time function based on continuous logistic growth. For management purposes, this model promotes proactive investments by quantitatively presenting the consequences of delayed interventions, thereby maximizing return on spending, especially for biological invasions that are in their early stages or even have not yet occurred locally (Ahmed, Hudgins, Cuthbert, Kourantidou, et al., 2022). Our model only requires a time series of cost estimates to project future cost patterns as well as the population dynamics of the target population. By relying solely on damage cost data, it avoids the need for detailed ecological or demographic inputs, making it both simple and broadly applicable across a wide range of non‐native species and contexts. In doing so, the temporal economic cost patterns can be used to retrace the population dynamics of non‐native species linked to impact, which are fundamentally governed by their intrinsic growth rates and carrying capacities. In the absence of data on management intensity based on observed damage, we assume a business‐as‐usual management scenario in our projections. In other words, we project damage if future management intensity will follow the same trends as in the past. In practice, however, escalating damage often stimulates more intensive interventions with possible threshold effects and discontinuities, meaning that actual costs could be lower than our projections. Projecting potential future damages up to 2050, we provide timescales for stakeholders to implement management strategies that address both current invasions and prepare for future threats. Given the need for cost efficiency in the presence of constrained management budgets, these dynamic projections can help optimize and prioritize investments for individual invasions. This straightforward model also permits projection of future cost dynamics based on initial cost data before rapid growth, as well as for future impacts of well‐established non‐native populations. We hypothesize that past and future damage–cost dynamics from non‐native species follow a consistent density‐dependent relationship with time, characterized by initially low costs that then rapidly escalate, with future projections possible even from limited early‐stage invasion‐cost data. However, density‐dependent relationships with costs will expectedly differ among species given their varying life‐history traits and impacted sectors. We also hypothesize that threshold time points along the cost curve and its underlying parameters (i.e., *C*_max_, γ, α) will be species‐specific, given differences in life‐history traits, environmental responses, and times since invasion. Finally, we hypothesize that our cost model will project impacts to increase into the future, even for late‐stage invasions, with the magnitude of changes varying depending on the current position of species along the cost curve.

## METHODS

### Continuous‐time logistic growth model

A general class of models for the population growth of a single species over continuous time can be expressed as follows:

(1) $$\frac{\mathrm{du}}{\mathrm{dt}}=\mathrm{uf}\left(u\right),u\left(0\right)=u_{0}$$

where *u*(*t*) represents the population density (e.g., number of individuals per unit area) as a function of time *t*, and *u*_0_ is the initial population density. The function *f*(*u*) represents the per capita growth rate, which typically depends on population density. For many species, especially at high population densities, *f*(*u*) decreases as *u* increases (known as *compensation*; Bradshaw & Herrando‐Pérez, 2023; Herrando‐Pérez et al., 2012), reflecting the ecological reality that growing populations are ultimately limited by an average, long‐term environmental carrying capacity *K*.

One of the simplest and most widely used models capturing this density feedback is the logistic growth model (Bradshaw & Herrando‐Pérez, 2023). In this model, the per capita growth rate decreases linearly with increasing population density, illustrating the competition for limited resources (e.g., food, space, or mates) that intensifies as population size grows. The per capita growth rate in the logistic model is given by

(2) $$f\left(u\right)=⍺\left(1-\frac{u}{K}\right)$$

where α > 0 is the maximum intrinsic growth rate under ideal conditions and no environmental constraints, and *K* > 0 is the environmental carrying capacity—the maximum population density that the environment can sustain without degradation or resource depletion. As population density approaches *K*, the growth rate declines to zero (i.e., resulting in temporal stability in population size).

The logistic growth model is described by

(3) $$\frac{\mathrm{du}}{\mathrm{dt}}=⍺u\left(1-\frac{u}{K}\right),u\left(0\right)=u_{0}$$

but can be written in a more convenient form if we introduce a variable to define the rescaled population density as *z* = *u*/*K*, where 0 < *z* < 1. The equation then becomes

(4) $$\frac{\mathrm{dz}}{\mathrm{dt}}=⍺z\left(1-z\right),z\left(0\right)=\frac{1}{\gamma }$$

which can be solved to express the rescaled population density at any time *t*:

(5) $$z\left(t\right)=\frac{1}{1+\left(\gamma -1\right)e^{-⍺t}}$$

where γ = *K*/*u*_0_ is an environmental scaling factor that represents the relationship between initial population density *u*_0_ and carrying capacity *K*, reflecting how environmental resources or limitations scale with initial population size. If α remains constant, carrying capacity becomes directly proportional to initial population density, indicating stable ecological conditions, such as consistent resource availability or habitat quality for specific species. In this case, growth slows as the population approaches *K* due to compensation. This shift moves the model from one where both *K* and *u*_0_ influence population dynamics to one where their ratio (γ) is the main driver. By focusing on γ, the model captures how environmental factors interact with initial population density to shape growth. An increase in γ reflects more abundant resources or optimal conditions, while a lower γ indicates resource limitation.

The logistic model effectively describes population growth in resource‐limited environments by balancing intrinsic growth potential and environmental constraints. Equation (5) shows that when population density is much smaller than carrying capacity (*u*_0_ ≪ *K*), the population grows exponentially. However, as *u*(*t*) approaches *K* (i.e., *z*(*t*) → 1), the growth rate slows, and the population stabilizes around *K*. This is a common feature in many ecological systems, where populations initially exhibit rapid growth when resources are abundant, but eventually stabilize at or near *K* (Brook & Bradshaw, 2006). Although widely applicable across species, it assumes a constant carrying capacity but does not account for environmental fluctuations, potential Allee effects (Courchamp et al., 1999) at low population densities, or dynamic factors influencing stationarity (Bradshaw & Herrando‐Pérez, 2023). The latter study demonstrated that logistic growth models are highly robust to non‐stationarity in *K*, including stochastic fluctuations and gradual declines, and resilient to other perturbations such as catastrophes. These models consistently detected density feedback in most cases, with only sustained population declines reducing robustness. Although variation in α can weaken model performance slightly, variability in *K* has little effect, supporting the suitability of this framework for our analysis.

Although other growth models also provide exact solutions to Equation (1), we focus on the continuous (cf. discrete) logistic growth model because it is simple, widely understood, and not prone to issues of parameter identifiability when tested against empirical data (Clark et al., 2010; Simpson et al., 2022). This is particularly important because failure to account for parameter identifiability can result in unreliable or imprecise parameter estimates, potentially leading to incorrect interpretations of the mechanisms involved (Clark et al., 2010). Moreover, its ability to describe the basic pattern of population growth, saturation, and stabilization makes it a baseline choice for many ecological applications.

For our study, this population framework underpins the cost models developed in the following section. However, we do not interpret the intrinsic growth rate (α) as a purely ecological parameter. Because our modeling focuses exclusively on damage costs, α should instead be regarded as a net accrual rate of damage costs under business‐as‐usual management. In other words, α captures how damages escalate through time given prevailing intervention and thus provides a baseline trajectory of impacts in the absence of enhanced management. We stress that this represents an upper bound trajectory because stronger‐than‐historical management responses would be expected to slow or reduce future cost accrual.

### Cost–density relationships

Previous studies have used cost–density curves to link the density of non‐native species with the costs incurred by socio‐ecological systems (e.g., Yokomizo et al., 2009). In many cases, managers assume that impact increases proportionally with invader density, especially when the exact density‐impact relationship is unknown (Elgersma & Ehrenfeld, 2011); however, ecological impacts often follow nonlinear patterns (Jackson et al., 2015). In practice, the connection between the ecological impacts of a non‐native species and its population density exhibits both linear and nonlinear patterns (e.g., Bradley et al., 2019; Finnoff et al., 2005; Laverty et al., 2017; Moroń et al., 2019; Nava‐Camberos et al., 2001). However, research that explicitly links these relationships to the monetary costs resulting from non‐native species impacts is scarce, albeit there have been some attempts to address the gap (Ahmed, Hudgins, Cuthbert, Haubrock, et al., 2022).

**FIGURE 1:**
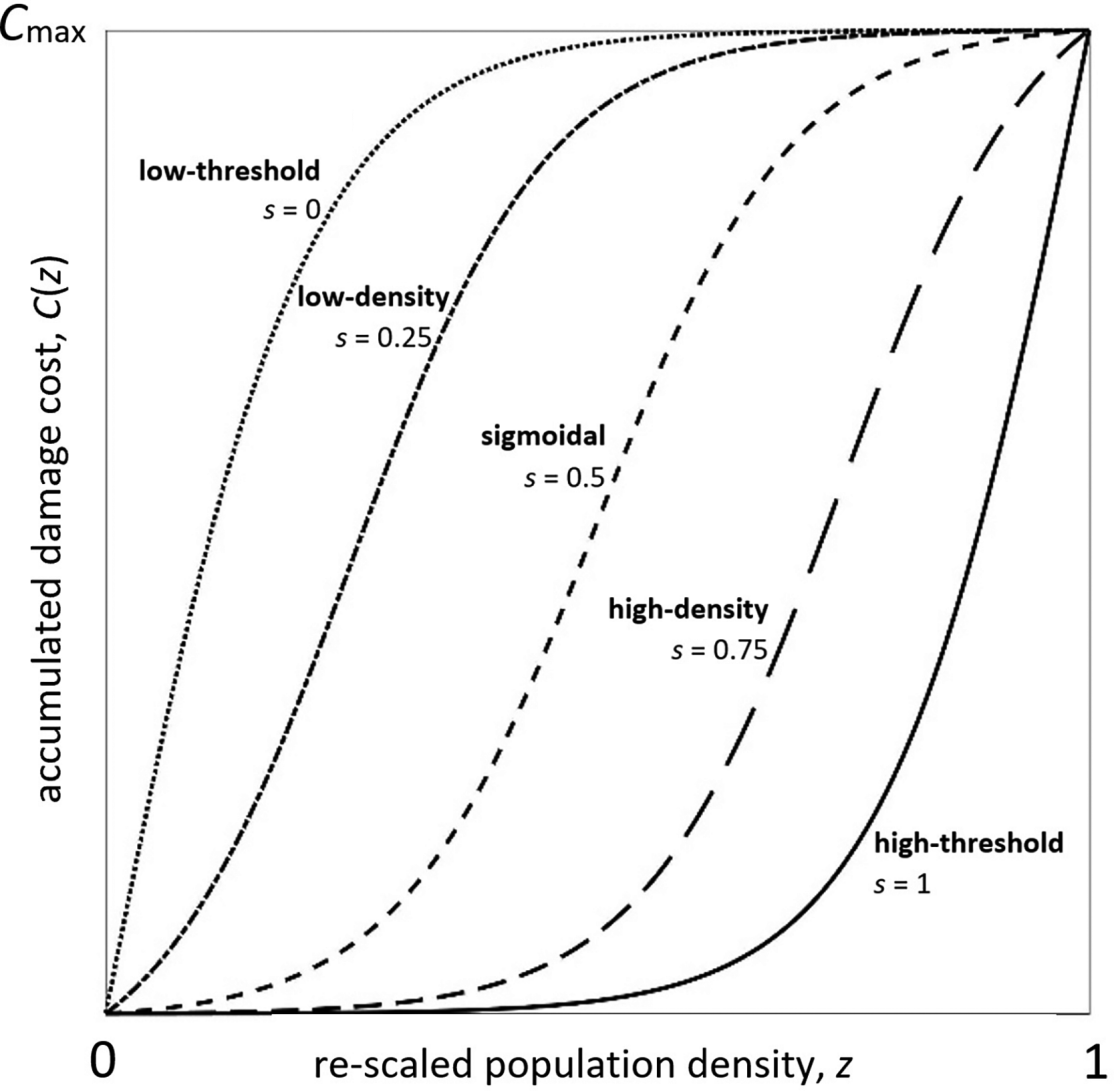
Illustration of the five types of cost–density curves: low‐threshold curve with shape parameter *s* = 0, low‐density curve with *s* = 0.25, sigmoidal curve with *s* = 0.5, high‐density curve with *s* = 0.75, and high‐threshold curve with *s* = 1. Adapted from Figure 2 in Ahmed, Hudgins, Cuthbert, Haubrock, et al. (2022).

To characterize the diverse forms of cost–density relationships—where the total incurred accumulated damage cost *C* depends on the (rescaled) population density *z*, we adopted a framework based on functional types proposed by (Yokomizo et al., 2009), expressed as

(6) $$C\left(z\right)=aC_{\max}\left(\frac{1}{e^{-10\left(z-s\right)}+1}-b\right),0<z<1$$

where

(7) $$a=\frac{1+e^{-c}}{1-b\left(1+e^{-c}\right)},b=\frac{1}{1+e^{10s}}\;\text{and}\;c=10\left(1-s\right)$$

and where *C*_max_ represents the maximum potential cost, and *s* is a shape parameter that defines the distinct types of cost–density relationships (Figure 1). For ease of presentation, two quantities *a* and *b* are introduced, which are expressed solely in terms of *s*, allowing the model to be written in a more compact form. This model is well‐suited to density–cost dynamics due to its use in evaluating ecological and economic impacts (e.g., Jackson et al., 2015; Roberts et al., 2018; Sofaer et al., 2018; Vander Zanden et al., 2017). It was specifically applied to capture the temporal dynamics of invasion‐related economic costs across several genera, revealing distinct types of cost curves and showing how variation in invasion duration, species' ecology, and data availability influence cost patterns and guide management strategies (Ahmed, Hudgins, Cuthbert, Haubrock, et al., 2022).

The parameter *C*_max_ assumes no further spatial spread of the non‐native species within the study area, implying that its current distribution reflects a stable ecological or climatic equilibrium (e.g., Aplin et al., 2011; Barnett, 2001). This assumption is reasonable for species that appear to have saturated their potential range, and from an intertemporal perspective, reaching this bound could reduce long‐term management costs by limiting the need for sustained control. Although annual damage costs can still accrue beyond this density, they are typically lower than during the phase of rapid population growth (Ahmed, Hudgins, Cuthbert, Haubrock, et al., 2022). As the population density stabilizes near its ecological limit, total costs approach a saturation point, asymptote at or near *C*_max_, potentially reflecting ecological or socioeconomic adaptation or habituation to the invader's presence within a given area; however, additional costs can still arise through range expansion or dispersal into new regions (Simberloff & Gibbons, 2004; Strayer et al., 2006). At that stage, the impact of additional population growth on costs diminishes, shifting the focus from preventing further spread to mitigating the ongoing negative economic and ecological impacts. We therefore treat *C*_max_ as “near” saturation, acknowledging that it does not imply cessation of costs but rather a practical asymptote where further annual cost increases are negligible relative to historical totals.

The relationship between the rescaled population density *z* of non‐native species and the damage costs they incur can be described broadly using five functional cost‐curve types, each reflecting a distinct pattern of cost accumulation as density increases (Figure 1). The low‐threshold cost curve shows a sharp rise in costs at low densities, followed by a plateau where costs remain consistently high despite additional population growth. The low‐density cost curve also exhibits an early escalation in costs, although less abrupt than the low‐threshold case. The sigmoidal cost curve exhibits a steep escalation in costs at intermediate densities. The high‐density cost curve delays escalation until populations reach relatively higher densities, after which costs rise progressively, while the high‐threshold cost curve is characterized by minimal cost accumulation at lower densities before experiencing a sharp surge once the population exceeds a critical density threshold.

### Temporal damage–cost dynamics

To link the damage cost *C* to time, we combined the logistic growth model (Equation 5) with the cost–density relationship (Equations 6 and 7):

(8) $$C\left(z\right)=aC_{\max}\left(\frac{1}{e^{-10\left(z-s\right)}+1}-b\right),z\left(t\right)=\frac{1}{1+\left(\gamma -1\right)e^{-⍺t}},0<z<1$$

with shape parameters *a* and *b* defined as in Equation (7). The damage cost function *C*(*z*) depends on the shape parameter *s*, while population dynamics are governed by the intrinsic growth rate α and the environmental scaling factor γ. By integrating ecological and economic dynamics, this temporal model illustrates how damage costs evolve as the population of non‐native species grows and stabilizes. It enables researchers and policymakers to project not only the immediate economic impacts of an invasion, but also how these costs will change over time. This approach is useful because it makes it possible to analyze trends in costs over time as a function of the growth of the species, helping to develop better informed, long‐term management strategies. By considering how population growth slows as the species approaches equilibrium, it helps in projecting the ongoing economic burden and guiding resource allocation for prevention and control.

### Identifying thresholds

We propose several thresholds for management along the damage cost curve to determine when non‐native species reach densities beyond which the economic and ecological consequences rise precipitously. The high‐density and high‐threshold damage‐cost functions can be normalized by introducing a rescaled cost variable $\hat{C}=C/C_{\max}$ which lies between 0 and 1, and expressed as

(9) $$\hat{C}\left(z\right)=\frac{a}{e^{-10\left(z-s\right)}+1},0<z<1,$$

with values $a=1+e^{-2.5},s=0.75$ in the case of a high‐density curve and *a* = 2, *s* = 1 in the case of a high‐threshold curve (Appendix S2). We define the density beyond which costs begin to escalate rapidly as when the rate of change of normalized cost with density is >1. To identify that density, we set $d\hat{C}/\mathrm{dz}=1$. Thus, the threshold density *z*_thresh_ for each case can be computed from Equation (9):

(10) $$z_{\text{thresh}}=s-\frac{1}{10}\log_{e}\left[5a-1+\sqrt{5a\left(5a-2\right)}\right].$$

For the high‐density curve, the threshold density is *z*_thresh_ ≈ 0.534, and for the high‐threshold curve it is $z_{\text{thresh}}=1-\frac{1}{10}\log_{e}\left(9+80\right)≃0.711$, occuring at time *t*_thresh_:

(11) $$t_{\text{thresh}}=-\frac{1}{⍺}\log_{e}\left[\frac{1}{\gamma -1}\left(\frac{1}{z_{\text{thresh}}}-1\right)\right].$$

The corresponding normalized costs are $\hat{C}\left(z_{\text{thresh}}\right)≃0.111$ for the high‐density curve and $\hat{C}\left(z_{\text{thresh}}\right)=1-\frac{2}{5}\sqrt{5}≃0.106$ for the high‐threshold curve (Equations 9 and 10). Thus, for each case, the accumulated damage cost at the threshold point is about 11% of *C*_max_, irrespective of the type of cost–density relationship. The detailed steps of this computation are in Appendix S3.

In terms of variables, *z* = *u*/*K*, so it follows that the true threshold density *u*_thresh_ is directly proportional to each species' carrying capacity *K*, given by *u*_thresh_ ≈ *z*_thresh_ *K*. Beyond this point, the marginal damage cost (i.e., the rate of change of accumulated damage cost with respect to population density; *dC*/*du*) exceeds the average cost per unit density across the full ecological range (*C*_max_/*K*). Ecologically, this represents a transition point: below *u*_thresh_, costs accumulate slowly, but beyond it, even small increases in invader density can drive disproportionately large increases in economic damage. The thresholds therefore mark the onset of rapid cost escalation and serve as a warning point for timely management intervention.

Consider the accumulated damage cost reaching a proportion ε of the long‐term potential cost *C*_max_. If we set $\hat{C}\left(z\right)=\varepsilon$ in Equation (9), the corresponding saturation population density *z*_sat_ is

(12) $$z_{\mathrm{sat}}=s-\frac{1}{10}\mathrm{lo}g_{e}\left(\frac{a}{\varepsilon }-1\right)$$

that occurs at time:

(13) $$t_{\mathrm{sat}}=-\frac{1}{⍺}\mathrm{lo}g_{e}\left[\frac{1}{\gamma -1}\left(\frac{1}{z_{\mathrm{sat}}}-1\right)\right],$$

(Appendix S3). To define the concept of “near” saturation, we set ε = 0.9, so that the density *z*_sat_ and the corresponding time *t*_sat_ are the point where damage costs reach 90% of their maximum value *C*_max_, determined from Equations (12) and (13). Similarly, “half” (mid) saturation occurs when ε = 0.5, where *z*_mid_ and *t*_mid_ correspond to 50% of *C*_max_. We use different subscripts to distinguish between these cases.

For the high‐density curve, the threshold densities are *z*_mid_ ≈ 0.735 and *z*_sat_ ≈ 0.910, whereas for the high‐threshold curve $z_{\mathrm{mid}}=1-\frac{1}{10}\log_{e}3\approx 0.890$ and $z_{\mathrm{sat}}=1-\frac{1}{10}\log_{e}\left(\frac{11}{9}\right)\approx 0.980$. In each case, the corresponding species densities are proportional to *K*, given as *u*_mid_ ≈ *z*_mid_ *K* and *u*_sat_ ≈ *z*_sat_ *K*. These density thresholds depend on the curve shape parameter of the cost–density curve (*s*), whereas the times at which these occur depend also on ecological parameters (α, γ) that govern the population dynamics of the species under consideration.

We also identified the inflection point of the cost–density curve, which marks the transition from an accelerating to a decelerating rate of increase in accumulated cost as density rises. This occurs at *z*_inflect_ = *s*, corresponding to a normalized cost of $\hat{C}\left(z_{\text{inflect}}\right)=a/2$. For the high‐density curve, the inflection point is *z*_inflect_ = 0.75, $\hat{C}\left(z_{\text{inflect}}\right)\approx 0.541$, occurring at time:

(14) $$t_{\text{inflect}}=-\frac{1}{⍺}\mathrm{lo}g_{e}\left[\frac{1}{3\left(\gamma -1\right)}\right].$$

Therefore, when the population reaches 75% of the carrying capacity, approximately 54% of *C*_max_ has already accumulated, after which the rate of cost escalation slows as the curve approaches saturation.

For the high‐threshold curve, the inflection point occurs at *z*_inflect_ = 1; because this lies outside the admissible domain 0 < *z* < 1, the curve remains in the accelerating phase across the full ecological range, with economic damages intensifying progressively at an ever‐steeper rate as density approaches carrying capacity. Unlike the high‐density curve, there is no intermediate stage of slowdown in cost escalation (Appendix S3).

### Model demonstration using randomly generated damage cost data

Before applying the model to empirical invasion costs, we first did a heuristic demonstration with randomly generated data. This example illustrates the damage–cost dynamics for two hypothetical non‐native species, showing in a controlled setting that the model can be fitted reliably even with limited early‐stage cost information. It also demonstrates how milestones along the cost trajectory (e.g., threshold, midpoint, and near saturation) can be identified, clarifying the model's functionality and its suitability for projecting long‐term cost dynamics in more complex, real‐world applications.

We randomly generated annual costs independently from a uniform distribution between US$0 and US$1 million for 10 consecutive years (time *t* = 0 to *t* = 9 years) and then cumulatively summed these to produce the input data for model fitting using Equation (8). As a demonstration, we only considered the high‐threshold cost–density curve with shape parameters *s* = 1 (Figure 1). For that scenario, we used the nonlinear regression function *fitnlm* in MATLAB to fit the model to the generated cost data and estimated the best‐fitting parameters: long‐term accumulated cost *C*_max_, environmental scaling factor γ, and intrinsic growth rate α. We evaluated the strength of the fit using root mean‐squared error (RMSE) and adjusted *R* ^2^.

We also consider a second scenario assuming the same *C*_max_ and γ, but with a reduced population growth (α/2). We identified indicative points along the damage cost trajectories, such as the threshold (point prior to rapid cost escalation), midpoint (when costs reach 50% of *C*_max_), and near saturation (when costs reach 90% of *C*_max_). We determined these points based on population density thresholds (Equations 10 and 12) and the ecological parameters α and γ (Equations 11 and 13).

Although the reliability of nonlinear model fitting depends on the quantity and quality of available data, we use 10 data to estimate three parameters. Although our model involves combined dynamical and cost–density equations, it is low dimensional (three parameters) and structurally identifiable, allowing stable fitting from few data. As a guideline, a minimum of three to five data per parameter is commonly regarded as acceptable when models are well specified and residuals are well behaved (Motulsky & Christopoulos, 2004). Accordingly, we used the same fitting approach across all cost–density curve types in Figure 1 in the empirical species‐level analyses (Table 1), which rely on similar or larger datasets.

**TABLE 1:** Number of independent years with recorded damage costs (*n*), period over which these costs were observed, best‐fitting cost–density relationship for each species, estimated long‐term accumulated cost (*C*_max_), environmental scaling factor (γ = 𝐾/𝑢_0_, where *K* = carrying capacity and *u*_0_ = initial population density), maximum intrinsic growth rate (α) with respective 95% confidence intervals in square brackets, and initial rescaled population density (*z*(0) = 𝑢_0_/*K* = 1/γ) of the five non‐native mammal species in Japan that we analyzed.

Species	*n*	Cost reporting period	Top‐ranked cost–density curve	Long‐term accumulated cost (US$ million, *C*_max_)	Environmental scaling factor (γ)	Intrinsic growth rate (year^−1^; α)	Initial rescaled population density *z*(0) = 1/γ
*Callosciurus erythraeus*	16	2001–2017	High threshold (*s* = 1)	1.17 [1.08, 1.27]	1.98 [1.80, 2.16]	0.217 [0.185, 0.249]	0.505 [0.463, 0.556]
*Herpestes javanicus*	9	2000–2017	High threshold (*s* = 1)	0.29 [0.13, 0.46]	1.15 [1.07, 1.24]	0.085 [≈ 0, 0.201]	0.868 [0.810, 0.935]
*Myocastor coypus*	18	2000–2017	High density (*s* = 0.75)	21.31 [20.61, 22.01]	2.05 [2.01, 2.09]	0.126 [0.118, 0.134]	0.488 [0.479, 0.498]
*Paguma larvata*	18	2000–2017	High threshold (*s* = 1)	83.38 [74.48, 88.28]	1.93 [1.88, 1.97]	0.151 [0.142, 0.161]	0.519 [0.508, 0.532]
*Procyon lotor*	18	2000–2017	High density (*s* = 0.75)	57.31 [54.40, 60.22]	2.93 [2.80, 3.05]	0.140 [0.130, 0.150]	0.342 [0.327, 0.357]

*Note*: Values shown are from the top‐ranked model for each species, selected from five alternative cost–density curves based on the lowest root mean‐squared error (RMSE) (in millions of US dollars) and/or lowest Akaike’s information criterion (AIC), together with the highest adjusted *R* ^2^. For *C. erythraeus*, *H. javanicus*, and *P. larvata*, the high‐threshold curve (*s* = 1) was top‐ranked, although for *M. coypus* and *P. lotor* the high‐density curve (*s* = 0.75) was most strongly supported. The full set of alternative model outputs, including all cost–density curves and associated fit statistics, is in Appendix S4. High adjusted *R* ^2^ reflect model fit to cumulative costs and should not be interpreted as predictive accuracy for annual cost variation.

### Data filtering and extraction

We extracted cost data from the *InvaCost* database version 4.1—the most comprehensive database of non‐native species costs—via the invacost R package (Leroy et al., 2022a, 2022b). We chose to restrict our analysis to Japanese data due to the high quality of cost records from this region. Although filtering to a single country reduced the cost entries included to those coming from a single publication, this set of cost records was the only example of consistent temporal cost reporting of non‐native species in the same location over time, highlighting the need for analogous studies in other countries to determine the generality of our findings and/or drivers of discrepancies across regions.

Although the *expandYearlyCosts* function in the invacost R package is typically applied to disaggregate, multiyear cost records into annual values, in Japan, all costs for the five focal mammal species were reported separately on an annual basis. Consequently, no cost expansion was required, and we analyzed the observed annual values directly. We considered the complete set of available annual cost records for our focal species in Japan up to 2022. The earliest cost entries begin in 2000 for all species except *C. erythraeus* (2001). Using the full dataset ensured that we included all reported costs in the analysis, although later records are more likely to be consistent in terms of valuation methods and sectoral detail.

Further filtering is typically applied to *InvaCost* data to ensure cost records are highly reliable and comparable across species. However, all Japanese cost entries for these species already had clear starting and ending years (per the “Probable_starting_year_adjusted”/“Probable_ending_year_adjusted” columns), non‐missing values in 2017 US dollars (per “Raw_cost_estimate_2017_USD_exchange_rate”), and were *observed* damage costs that are considered to be highly reliable (per “Type_of_cost_merged”, “Implementation”, and “Method_reliability_refined” columns, respectively).

We only modeled *observed* accumulated damage costs over time. Therefore, we did not include any earlier costs that went unreported in our analysis. As a result, our model probably underestimates the true long‐term economic impact of these species. However, because costs tend to rise slowly in the early stages of invasion and escalate sharply only after reaching a threshold density, these missing early costs are likely small in magnitude relative to the peak costs. Thus, although our estimates might slightly understate total accumulated costs, the overall trajectory and main escalation patterns are unlikely to be qualitatively affected. Because our dataset contained only single‐year annual costs, we did not apply expansion functions, thereby avoiding assumptions about intra‐period cost distributions. Moreover, this temporal pattern—where annual costs start low, peak, and then decline, forming a bell‐shaped curve—has been observed for several non‐native species (e.g., *Aedes* spp.; Ahmed, Hudgins, Cuthbert, Kourantidou, et al., 2022), supporting the assumption that early‐stage costs typically contribute only a small proportion of long‐term damages.

Following these filters, we identified five mammal species: *C*. *erythraeus*, *H*. *javanicus*, *M*. *coypus*, *P*. *larvata*, and *P*. *lotor*. Taxonomy follows the GBIF Backbone Taxonomy (GBIF Secretariat 2023), as adopted by the *InvaCost* database, to ensure consistency with the source data. Alternative classifications place *H. javanicus* within the genus *Urva* (Veron & Jennings, 2017). We re‐checked database entries to ensure concordance in recorded cost timing and the timing noted in the associated report and corrected any discrepancies. We aggregated annual cost records by year and species and calculated cumulative costs from the first year of cost records by each species to the final year of their costs.

We took the year of first record for each species from the *Standardizing and Integrating Alien Species* database; SinAS v2.4.1 (Seebens et al., 2020), except for *P. larvata*, which was listed as pre‐19th Century. This exception reflects historical uncertainty about the species' origin in Japan. During the Edo period (1603–1868), illustrations appeared to depict civet‐like animals, leading some to think the species was native. However, recent genetic studies have confirmed that *P. larvata* is non‐native (Endo et al., 2020). In fact, the exact date of introduction is unknown, but it is now widely thought that the civet was brought to Japan from Taiwan sometime in the 1930s or 1940s. The earliest reliable field record of civet capture dates to 1943 in Shizuoka (Nawa, 1965), supporting the conclusion that *P. larvata* should be considered a 20th‐Century introduction.

### Model fitting

To model and project the damage costs associated with the five non‐native mammal species, we used observed damage cost data expressed in millions of US dollars (2017 value) to fit the damage cost model based on the continuous‐time logistic growth model (Equation 9). We analyzed each species over a period defined by the first and last reported years of damage cost data after the database had been filtered. We designated the starting year of the time series as *t* = 0, allowing for a consistent relative timeline for each species (e.g., *C. erythraeus* from 2001 to 2018, or *t* = 0 to *t* = 17). The damage cost model is parameterized by the maximum accumulated cost (*C*_max_), the environmental scaling factor (γ), and the intrinsic growth rate (α). The initial rescaled population density is *z*(0) = 1/γ and is therefore not an independent parameter but determined directly from the estimated value of γ. We estimated these population dynamical parameters by fitting the model directly to the damage cost time series. As such, the only required inputs were the damage cost data, the timing of reported costs, and a prior specification of the cost–density relationship (Figure 1). We did not fit the density component of the function to species‐specific population estimates over time, as is typically done in discrete‐time logistic models.

We used the nonlinear regression tool *fitnlm* in MATLAB to fit the cost data and determine the best‐fit model parameter set (*C*_max_, γ, α) for each species, while considering each of the four cost–density relationships separately. For all species, we fitted five candidate cost–density relationships (low threshold, low density, sigmoidal, high density, and high threshold; Figure 1) and identified the top‐ranked model based on a robust statistical criterion: lowest RMSE (in millions of US dollars), Akaike’s information criterion (AIC), and highest adjusted *R* ^2^ (Appendix S4). Because all model types estimate the same number of parameters, AIC here serves primarily as a complementary, parameter bias‐corrected, likelihood‐based measure to compare relative support among models, rather than as a penalty for overfitting.

Cumulative cost data are inherently autocorrelated, because each value necessarily contains all previous years. This is a structural property of cumulative trajectories rather than a model artifact, so it does not bias parameter point estimates, nor does it compromise relative comparisons across candidate models that are all fitted to the same cumulative data. However, it can underestimate standard errors and inflate goodness‐of‐fit statistics such as adjusted *R* ^2^, so the confidence intervals reported for parameters (e.g., *C*_max_, γ, α) and projected trajectories should be interpreted cautiously as conservative bounds rather than precise measures of uncertainty. This treatment is consistent with previous studies that have explicitly modeled cumulative costs or impacts using similar frameworks (Ahmed, Hudgins, Cuthbert, Haubrock, et al., 2022; Cuthbert et al., 2021; Haubrock et al., 2022; Soto, Ahmed, et al., 2023).

We used the model fitting to project damage costs for each species from the starting year of observed data up to 2050. We generated projections with 95% confidence regions to account for uncertainty. For 2050, we reported both the projected cost and the upper conservative bound of the future damage cost. For each species, we identified three indicative points along the damage cost trajectory: (1) the threshold where accumulated costs begin to rise rapidly, (2) the midpoint at which accumulated costs reach half of the potential maximum (0.5*C*_max_), and (3) the near‐saturation point, at which accumulated costs reach 90% of their maximum value (0.9*C*_max_). These estimates provide insights into the temporal dynamics of cost growth, while also highlighting time points for optimum management intervention.

## RESULTS

### Model demonstration using randomly generated damage cost data

**FIGURE 2:**
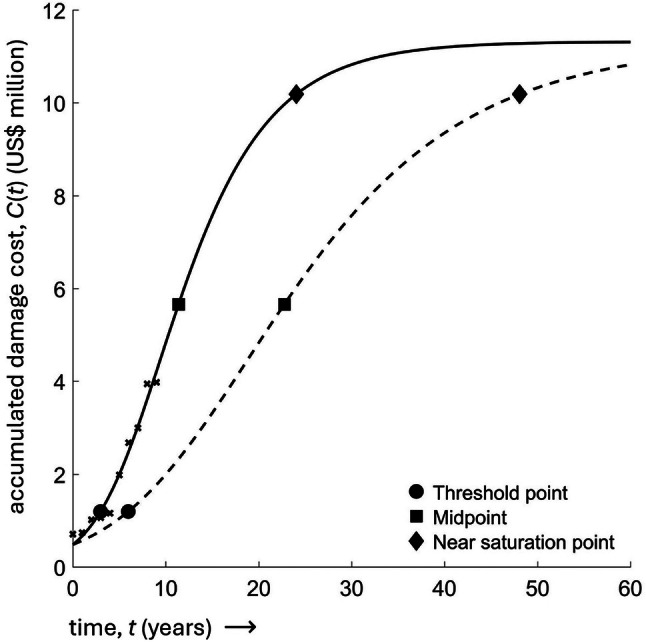
A heuristic example illustrating the damage–cost dynamics for two hypothetical non‐native species. The damage cost model (Equation 9) is fitted to cumulative annual costs that are randomly generated over 10 years from *t* = 0 to *t* = 9 (✖). Each annual cost is independently drawn from a uniform distribution ranging from US$0 to US$1 million. For case 1 (solid curve), the estimated best‐fitting parameters are long‐term accumulated cost *C*_max_ = US$11.32 million, environmental scaling factor γ = 1.62, and intrinsic growth rate α = 0.142, with strong goodness of fit quantified by a root mean‐squared error = 0.2557, adjusted *R* ^2^ = 0.9607. In another scenario, case 2 (dashed line) assumes the same *C*_max_ and γ, but with α halved (0.071), resulting in a slower cost accumulation. Salient time points are marked along the curves: the threshold (point prior to rapid cost escalation; ●), midpoint (costs reach 50% of *C*_max_; ■), and near saturation (costs reach 90% of *C*_max_; ◆). This shows how time milestones can be determined using early‐stage cost data, without requiring prior knowledge of their values.

Long‐term damage–cost dynamics could be effectively projected from early‐stage impacts from invasions using our model (Figure 2). This analysis shows differences in the damage‐cost escalation dynamics between the two hypothetical scenarios. In case 1 (solid curve), the damage cost reached US$4.22 million at *t* = 9 years, with a long‐term cost projection rising to *C*_max_ = US$11.32 million, amounting to an additional US$7.10 million (168.4%) in damages over time. Critical time points occur early: The threshold point (US$1.19 million) is reached at 2.98 years, the midpoint (US$5.66 million) at 11.38 years, and near saturation (US$10.18 million) at 24.05 years. This delineates a period of rapid cost escalation of 21.07 years (i.e., duration between threshold and near‐saturation times).

In contrast, case 2 (dashed curve) examines the effect of halving α to 0.071, while keeping *C*_max_ and γ the same. As a result, curbing the rate of population growth delays cost escalation, with the threshold now occurring at 5.96 years, the midpoint at 22.76 years, and near saturation at 48.10 years, with the same respective damage‐cost estimates as in case 1. Consequently, the rapid cost escalation period doubles in duration to 42.14 years.

### Cost–density relationships

The high‐density curve (*s* = 0.75) was top‐ranked for *M. coypus* and *P. lotor*, and the high‐threshold curve (*s* = 1) best described *C. erythraeus*, *H. javanicus*, and *P. larvata* (Table 1; Appendix S4). Both curve types indicate relatively low costs at low population densities, but with distinct damage–cost dynamics. Under the high‐density curve, costs increased progressively with density and reached an inflection point at 75% of carrying capacity, by which stage about 54% of the maximum accumulated cost (*C*_max_) had been realized. Beyond this point, the rate of escalation slowed as the curve approached saturation. By contrast, under the high‐threshold curve, costs remained minimal until populations reached much higher densities, at which point they surged sharply. With no inflection point within the ecological range (0 < *z* < 1), costs under this curve remained in the accelerating phase throughout, intensifying progressively at an ever‐steeper rate as density approached carrying capacity (Figure 1; Equation 10). This demonstrates that although early invasion stages involve relatively low costs for all species, the escalation phase differs—progressive but slowing in *M. coypus* and *P. lotor*, whereas delayed and abrupt in *C. erythraeus*, *H. javanicus*, and *P. larvata*.

### Long‐term damage costs and population dynamics

The long‐term accumulated cost *C*_max_ representing the maximum economic impact varied substantially across species. *P. larvata* had the highest *C*_max_ at US$83.38 million, and *H. javanicus* had the lowest *C*_max_ at US$0.29 million. Other species, such as *P. lotor* (US$57.31 million) and *M. coypus* (US$21.31 million), also had substantial economic impacts, with *C. erythraeus* at US$1.17 million (Table 1).

The environmental scaling factor γ (= *K/u*_0_) provides insights into how environmental conditions influence population dynamics, particularly in relation to resource availability. *P. lotor* had a high γ of 2.93, indicating its strong capacity to exploit available resources under the given conditions. Similarly, *C. erythraeus* (γ = 1.98) and *P. larvata* (γ = 1.93) demonstrated similar potential in their respective environments. However, although high γ indicates favorable conditions for growth, it does not on its own imply broad ecological adaptability, which depends on other factors such as behavioral plasticity and habitat generalism. In contrast, species such as *H. javanicus* (γ = 1.15) demonstrate lower environmental scalability, potentially reflecting the isolation of the species to two remote islands.

The maximum intrinsic theoretical growth rate (α) also varied according to the species. *C*. *erythraeus* had the highest intrinsic growth rate (0.217), followed by *P*. *larvata* (0.151), *P*. *lotor* (0.140), *M*. *coypus* (0.126), and *H*. *javanicus* (0.085). The higher this rate, the more abrupt the cumulative costs, which highlights a relationship between the growth rate of the non‐native species and the slope of the cumulative cost function. If the damage cost data already reflect periods during which management interventions were active (e.g., population control or containment), then α is understood as a *realized* or *net* growth rate, that is, the observed population growth under the influence of both ecological conditions and ongoing control measures. In such cases, α captures the combined effects of the population dynamics and existing management actions, rather than a purely unmanaged growth potential.

**FIGURE 3:**
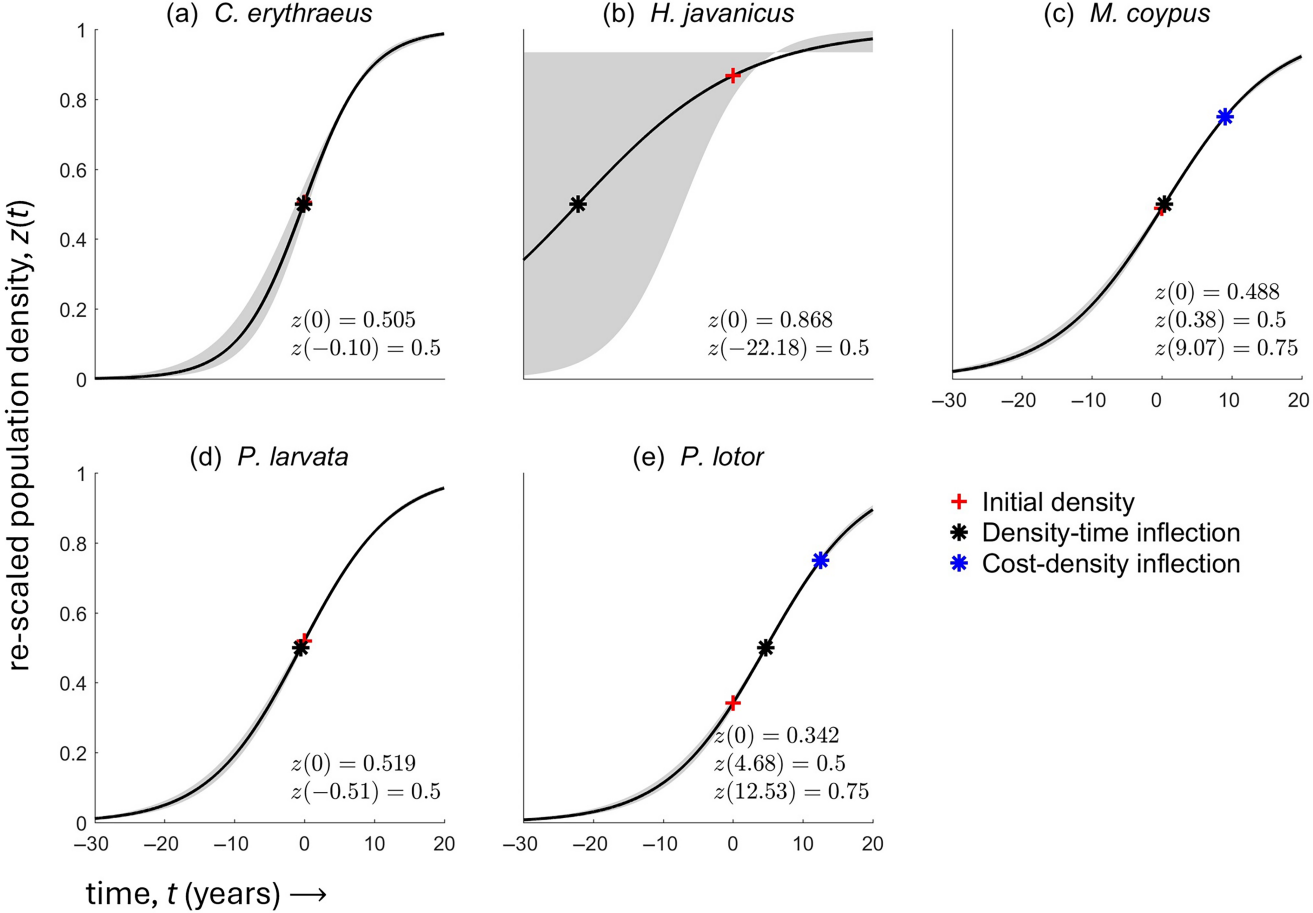
Rescaled population density plots for the five non‐native mammal species from Japan, modeled using the logistic growth model (Equation 5). The rescaled density for each species at the time of first reported cost (*t* = 0) is *z*(0) = 1/γ = *K*/*u*_0_; where *K* = carrying capacity and *u*_0_ = initial population density; Equation (4) (✛). Carrying capacity *K* is reached as the population *z*(*t*) approaches 1. The density–time inflection (✱) indicates a shift from an increasing to a decreasing rate of population density that occurs at half the carrying capacity, or equivalently, *z* = 0.5 at time t*_inflect_ = (1/α)log_*e*_(γ − 1). For species following the high‐density curve (*Myocastor coypus*, *Procyon lotor*), we also mark the cost–density inflection (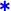) where costs transition from accelerating to decelerating, at *z* = 0.75 and time *t*_inflect_ (Equation 14). The other species follow a high‐threshold curve, and thus have no inflection within 0 < *z* < 1. The 95% confidence region is shown (shaded area), plotted using Equation (5), with the upper and lower confidence limits of α and γ provided in Table 1. For visualization, the time axis begins 30 years before the first cost is reported for each species.

The rescaled initial density, *z*(0) = *u*_0_/*K* = 1/γ, represents the proportion of the carrying capacity (*K*) occupied by the initial population. *P. lotor* had the lowest value (0.342), with density <0.5 *K* at the time of first cost reporting, and *H. javanicus* had the highest (0.868), indicating a population closest to its maximum sustainable size. We found intermediate values for *M*. *coypus* (0.488), *P*. *larvata* (0.519), and *C*. *erythraeus* (0.505) (Table 1; Figure 3).

The top‐ranked cost–density curve achieved high goodness of fit across all respective species, with consistently high adjusted *R* ^2^ and low RMSE (Appendix S4). Although these high adjusted *R* ^2^ confirm that the model captures cumulative cost trajectories well, they should be interpreted with caution because they do not indicate explanatory power for the more variable year‐to‐year costs (Weisberg, 2014). Species such as *C. erythraeus*, *M. coypus*, *P. larvata*, and *P. lotor* (adjusted *R* ^2^ > 0.995) therefore have reliable cumulative cost projections. In contrast, *H. javanicus* had a lower adjusted *R* ^2^ (0.914), reflecting noisier or incomplete data and higher variability in cost reporting, compounded by its smaller sample size. Confidence intervals for *C*_max_, γ, and α (Table 1) further illustrate precision in parameter estimates: Narrow intervals for *P. larvata* and *C. erythraeus* indicate high certainty, while broader intervals such as for *H. javanicus* highlight greater uncertainty and the need for additional cost records to refine projections.

The population density trajectories in Figure 3 illustrate how species‐specific growth dynamics can inform ecological strategies and potential management interventions. Species with higher intrinsic growth rates (α), such as *C. erythraeus* (α = 0.217), demonstrate rapid potential population growth, quickly saturating their environments.

Higher γ (e.g., *P. lotor*: γ = 2.93) imply populations starting from a lower baseline relative to *K*. In contrast, species with lower γ (e.g., *H. javanicus*: γ = 1.15) begin with densities closer to their carrying capacities, potentially reflecting stable population dynamics constrained by *K*.

For *C. erythraeus* and *P. larvata*, the density–time inflection occurred at roughly the same time that the first cost was reported, indicating that the earliest recorded impacts coincided with populations shifting from exponential growth to a slower, density‐regulated phase (Figure 3). By contrast, for *H. javanicus*, the density–time inflection preceded the first cost record, suggesting a lag between population dynamics and the point at which economic impacts were first documented.

The cost–density inflection point applies only to *M. coypus* and *P. lotor*, the two species characterized by a high‐density curve. For *M. coypus*, the sequence of events shows the first cost being reported at approximately the same time as the density–time inflection and before the cost–density inflection, beyond which the rate of cost escalation slows. For *P. lotor*, these three points occur in succession: the first cost coincides with the density–time inflection, followed later by the cost–density inflection. This ordering highlights the timing of events in species that follow a high‐density cost curve; impacts are reported earlier in the population trajectory, but prior to the slowdown in cost escalation.

### Thresholds

Our analysis revealed variability in the timing of escalation of economic impact across the five non‐native mammal species (Table 2), reflecting species‐specific differences in ecological dynamics, ecological traits, and the associated economic costs, despite similarities in the form of the underlying cost curve. Assuming a business‐as‐usual management, a few common trends emerged regarding the timing of threshold densities, the speed of cost escalation, and the opportunities for further management interventions.

**TABLE 2:** Year of first species record as listed in the *Standardising and Integrating Alien Species* SinAS database v2.4.1; except for *P. larvata*, where the year is reported in Nawa (1965). Main times along the damage cost curve of the five non‐native mammals in Japan, computed from Equations (11) and (13), including the threshold time (*t*_thresh_), beyond which damage costs begin to escalate rapidly, and the time to half saturation (*t*_mid_) and near saturation (*t*_sat_), when accumulated costs reach 50% and 90% of the potential maximum cost (*C*_max_), respectively. These times are measured from the year of the first species record. For each milestone, we report both the point estimate (from the best fit parameters) and the range obtained by accounting for the 95% confidence intervals of α and γ (Table 1), with the earliest times occurring at (α_max_, γ_min_) and the latest times at (α_min_, γ_max_). We also present the duration of rapid cost escalation (*t*_sat_ − *t*_thresh_, in years) and the period of rapid cost escalation (the corresponding calendar year interval), with both point estimates and ranges.

Species	Year of first record	Threshold time (years; *t*_thresh_)	Time to half saturation (years; *t*_mid_)	Time to near saturation (years; *t*_sat_)	Duration of rapid cost escalation (years; *t*_sat_ − *t*_thresh_)	Period of rapid cost escalation
*Callosciurus erythraeus*	1935	70.05	75.54	83.81	13.75	2005–2019
		[68.72, 71.67]	[73.50, 78.11]	[80.71, 87.82]	[12.00, 16.15]	[2004–2023]
*Herpestes javanicus*	1910	78.45	92.49	113.68	35.22	1988–2024
		<87.28		>96.12	>14.86	
*Myocastor coypus*	1939	62.44	69.44	79.66	17.22	2001–2019
		[62.07, 62.86]	[68.67, 70.35]	[78.31, 81.30]	[16.24, 18.44]	[2001–2020]
*Paguma larvata*	1943	62.45	70.32	82.19	19.74	2005–2025
		[61.81, 63.13]	[69.21, 71.52]	[80.36, 84.17]	[18.55, 21.03]	[2005–2027]
*Procyon lotor*	1962	43.64	49.96	59.19	15.55	2006–2021
		[42.81, 44.57]	[48.71, 51.38]	[57.32, 61.31]	[14.51, 16.74]	[2005–2023]

*Note*: For *H. javanicus*, the small sample size (*n* = 9; Table 1) leads to parameter uncertainty such that the 95% confidence intervals for milestone times are not finite. We therefore report point estimates with one‐sided bounds (shown as < or >) where applicable.

*Callosciurus erythraeus* had the shortest duration of rapid cost escalation, with costs rising sharply over just 13.75 years (2005–2019) following a threshold time of 70.05 years. This suggests that once economic impacts began to accelerate, they did so quickly, highlighting the urgency of early intervention for such species. In contrast, *H. javanicus* had a similar threshold time of 78.45 years, but its rapid cost escalation lasted longer at 35.22 years (1988–2024), making it the slowest escalating species in our dataset, the limited sample size available for this species notwithstanding.

*Myocastor coypus*, *P. larvata*, and *P. lotor* had intermediate patterns of cost escalation. *M. coypus* had a threshold time of 62.44 years and a 17.22 year escalation phase (2001–2019), indicating a moderate pace of impact intensification. Meanwhile, *P*. *larvata* exhibited a near identical threshold time (62.45 years), but a slightly longer escalation period of 19.74 years (2005–2025). In contrast, *P. lotor* had the shortest threshold time of 43.64 years, indicating that costs began escalating sooner after introduction than for other species. Its escalation period lasted 15.55 years (2006–2021), comparable in duration to *M. coypus* but shorter than *P. larvata*.

### Future projections of damage costs

**FIGURE 4:**
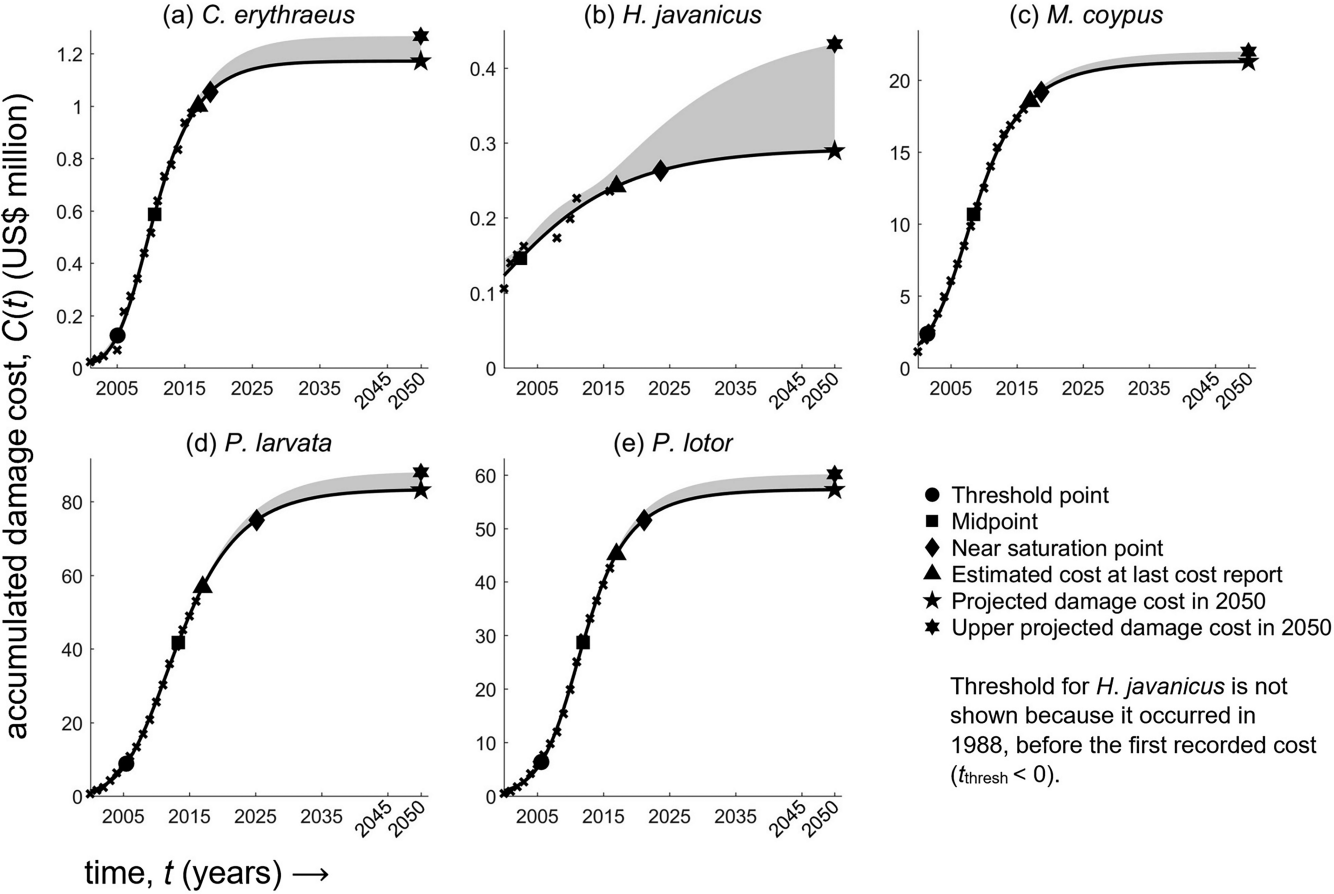
Projected damage costs over time for five non‐native mammal species in Japan (black curves), obtained from fitting the damage cost model (Equation 9) against observed cost data (✖). Markers along the cost curves indicate the threshold time (*t*_thresh_; ●) beyond which damage costs escalate rapidly, the midpoint (*t*_mid_; ■) when accumulated costs reach half the potential maximum cost (*C*_max_), and the time to near saturation (*t*_sat_; ◆) when costs reach 90% of *C*_max_. For *Herpestes javanicus*, the threshold occurred in 1988, prior to the first recorded cost in 2000 (i.e., with *t*_thresh_ < 0). We do not present this threshold because the damage cost curve only covers the period from the year when costs were first recorded. This illustrates a detection and reporting lag, where accelerated impacts likely began before they were documented, effectively rendering opportunities for timely management obsolete. Other markers include the estimated cost as per the fitted damage‐cost model (black curves) when the last recorded cost was reported (▲), and the projected damage cost in 2050 (★). We only consider the upper 95% confidence region (shaded area) whose upper bound demarcates the temporal cost estimates, used to estimate the 2050 upper conservative potential cost (✶). For illustrative purposes, the vertical cost axis is scaled independently for each species according to its cost magnitude.

Among the species we examined (Figure 4), *P. larvata* had the most pronounced absolute increase in economic costs, reflecting its continued period of rapid cost escalation (Table 2) and its high projected costs to 2050 (Table 3). Assuming management follows the prevailing trend, the projected cost for this species is projected to rise from an estimated US$56.79 million to US$83.18 million (a 46.47% increase). The upper confidence limit suggests an even greater potential rise of 54.94%, reaching US$87.99 million by 2050. Similarly, *P. lotor* is expected to experience a substantial cost increase, with economic impacts growing 26.68%, from US$45.18 million to US$57.23 million. The upper confidence limit suggests a possible 33.05% increase to reach US$60.11 million.

**TABLE 3:** Projected economic costs for five non‐native mammal species in Japan based on the fitted damage cost model.

Species	Estimated cost at the time of the last cost report ($US millions)	Projected cost for 2050 ($US millions)	Projected cost using the upper confidence limit ($US millions)	Percentage increase (%)	Percentage increase (%) (upper confidence limit estimate)
*Callosciurus erythraeus*	1.00	1.17	1.27	17.08	26.57
*Herpestes javanicus*	0.24	0.29	0.43	19.42	78.40
*Myocastor coypus*	18.53	21.28	21.97	14.83	18.55
*Paguma larvata*	56.79	83.18	87.99	46.47	54.94
*Procyon lotor*	45.18	57.23	60.11	26.68	33.05

*Note*: The estimated costs at the time of the last recorded report, the projected costs for 2050, and the upper limit of the 95% confidence interval as an upper conservative bound of the future cost. Percentage increases in costs from the last report to 2050 are presented, including those based on the upper bound estimates.

In contrast, *C. erythraeus* and *H. javanicus* are projected to have more moderate cost increases. The former is estimated to have a 17.08% rise from US$1.00 million to US$1.17 million, with an upper CL of 26.57% to reach US$1.27 million. *H. javanicus*, although having a relatively lower baseline increase of 19.42% (from US$0.24 million to US$0.29 million), has a substantially larger upper bound projection of 78.40%, indicating an estimate of US$0.43 million, suggesting potential underestimation of its future impact and greater uncertainty likely attributed to a low sample size.

*Myocastor coypus* followed an intermediate trajectory, with costs expected to rise 14.83% (from US$18.53 million to US$21.28 million). The upper confidence limit indicated an increase of 18.55%, to reach US$21.97 million. Overall, these projections highlight the varying rates of economic escalation among species, with *P. larvata* and *P. lotor* having the most substantial increases, while *C. erythraeus* and *H. javanicus* have more moderate but still important growth. The substantial upper bound increases, particularly for *H. javanicus*, reinforce the importance of considering uncertainty in future cost projections. This uncertainty arises from the range of the confidence intervals in the estimated parameters (Table 1). Species such as *H. javanicus*, with relatively wide intervals, yield highly variable projections, whereas species with narrower intervals (e.g., *M. coypus*, *P. larvata*) produce more constrained projections.

## DISCUSSION

Our mathematical models projecting future invasion costs leveraged economic impact data for five candidate non‐native mammals in Japan and incorporated both population and cumulative cost dynamics over time. Cost trajectories for all these non‐native species were well represented by the logistic growth model and conformed to a high‐threshold cost–density dynamic, with variable cost magnitudes, intrinsic growth rates, and environmental scaling in relation to resource availability. All the species we examined already surpassed the threshold year beyond which rapid growth in economic costs ensues, with most also recently surpassing the year of near saturation where >90% of costs have been incurred. Nevertheless, although these invasions can all be considered at a late stage based on their cost dynamics, our model still projects substantial increases of up to 78% by 2050 and absolute sums in the tens of millions of dollars. Moreover, we demonstrate that it can be used where only early‐stage cost data are available (i.e., before attaining half the long‐term cost) to project future dynamics. Our model thus presents a framework to characterize non‐native mammal populations and their cost dynamics to make future projections, while identifying threshold times for effective management actions before costs escalate rapidly. We found that periods of rapid cost escalation are preceded by extensive lag times when population densities and impacts of non‐native mammals are low—even if time lags to impact last centuries after introduction, cost escalation to maxima can occur within a few decades.

Slight differences in cost–density relationships notwithstanding (see below), these results support the notion that time lags can precede profound ecological and socioeconomic “surprises”, whereby sudden changes in non‐native populations can drive substantial impacts. This reinforces the need for applying the precautionary principle in management, requiring a perceptive paradigm shift from reactivity to proactivity, because long periods of benign effects can be poor surrogates for future impacts (Crooks, 2005). At the same time, apparent threshold patterns in cost trajectories could partly reflect socioeconomic perception and reporting lags (Crooks, 2005), whereby damages accumulate unnoticed or unrecorded until institutional or economic triggers result in sudden recognition and large reported costs. The patterns could inform timely management actions and future impact quantifications for these species in their so‐far uninvaded areas, as well as for other closely related non‐native species with similar economic impacts.

### Model demonstration using randomly generated damage cost data

Although the example cases are based on long‐term observed cost dynamics (i.e., multi‐decadal) and are therefore retrospective, we heuristically demonstrated that future cost trajectories can be projected with only early‐stage (i.e., sub‐decadal) cost data. This underpins the applicability of our approach for managers, where future projections can be made even using initial impact reporting with different assumptions for underlying parameters. This further highlights that early management actions that aim to control population growth can influence ecological and economic outcomes, particularly when aiming to reduce the midterm damage costs of non‐native species. Even with randomness in the input cost data, the model reliably captures the future damage–cost dynamics, and moreover, indicative time milestones such as threshold, midpoint or near‐saturation points can be identified from early‐stage cost data alone. Thus, this offers a robust framework for anticipating critical ecological thresholds and prioritizing proactive strategies. While the long‐term cost remains unchanged by design among the two scenarios considered, early management intervention effectively gives practitioners more time to mitigate intermediary damage impacts. Future research is needed to enable our modeling framework to be applied to empirically measured impacts at early invasion stages to validate cost trajectories as impacts subsequently accumulate.

### Cost–density relationships

Our analyses showed two distinct forms of cost–density dynamics: *M. coypus* and *P. lotor* were best described by the high‐density curve, while *C. erythraeus*, *H. javanicus*, and *P. larvata* followed the high‐threshold curve (Appendix S4). For high‐density species, costs rose progressively and exhibited an inflection point at 75% of carrying capacity, when about 54% of the maximum accumulated cost (*C*_max_) had already been reached. Beyond this point, escalation slowed as costs approached saturation. By contrast, for high‐threshold species, costs remained minimal across most of the density range and then surged abruptly at higher densities, with no inflection point within the ecological domain. This resulted in continuous acceleration of costs right up to carrying capacity.

These curve types capture different bioeconomic processes. For high‐threshold species, low‐density populations can remain ecologically inconspicuous or confined to natural environments until they expand into agricultural or urban areas, at which point costs escalate steeply—consistent with lag times, sleeper populations, or abrupt shifts in per capita impacts once density reaches a sufficient magnitude that allows interaction with agricultural or urban systems (Bradley et al., 2019; Sofaer et al., 2018; Soto, Ahmed, et al., 2023; Spear et al., 2021). For high‐density species, impacts accumulate earlier and more gradually, reflecting direct and continuous pressure on ecosystems and resources. The inflection point highlights that marginal impacts diminish once populations near carrying capacity, even though total costs remain high. Economically, thresholds correspond to ~10% of *C*_max_ (onset of rapid escalation), whereas inflection points represent ~54% of *C*_max_ (onset of slowdown). Together, these provide quantifiable benchmarks for management intervention.

The reasons for these contrasting dynamics might be explained by environmental heterogeneity within the landscape. Landscapes consist of natural environments (e.g., forests and rivers) and anthropized areas (e.g., agricultural fields, human settlements) where economic damage can occur, and where non‐native mammals can thrive (Biancolini & Rondinini, 2025). High‐threshold species likely reflect delayed impacts linked to spread into anthropized areas, whereas high‐density species exert continuous pressure even at intermediate densities. These five species mainly depend on resources in the natural environment for food and shelter, although they also feed on agricultural crops (Ohdachi et al., 2018), resulting in potential interplays between the natural and anthropized environment with respect to cost accumulation. Indeed, in Japan, there is a high proportion of natural areas (~70% forest cover) (FAO, 2020), so to become widespread, these species need to invade natural environments successfully before feeding back into human environments (and vice versa). In several modified areas, Pallas squirrels, raccoons, and civets depend on human subsidies (Kozakai et al., 2018; Tsukamoto & Suda, 2012; Yoshida et al., 2009), raccoons and civets settle in houses (Ikeda et al., 2004; Iwama & Kaneko, 2019), and animals generally become bolder, all of which increase opportunities for economic damage.

These cost dynamics can also be examined in the context of management interventions, as some species such as the small Indian mongoose have been successfully eradicated from parts of Japan. Following a long‐term intensive trapping program initiated in 2000, this species was declared eradicated from Amami‐Oshima Island in 2024, after population estimates declined from >6000 to near‐zero (Barun et al., 2011; Fukasawa, Hashimoto, et al., 2013). This eradication not only resulted in the recovery of native species (Fukasawa, Miyashita, et al., 2013; Watari et al., 2013) but is also expected to lead to a complete reduction in future damage costs in these fully managed areas (Watari et al., 2021; Yamada & Sugimura, 2004). Therefore, although our model operates at the national scale as applied here, separate submodels would be needed to capture local management successes, particularly at the scale of smaller islands.

Our results reveal two escalation pathways. High‐threshold species showed delayed but abrupt cost surges once populations exceeded critical densities, and high‐density species exhibited earlier, progressive cost accumulation that eventually slowed beyond the inflection point. These differential cost magnitudes and population density trajectories illustrate the importance of understanding species‐specific growth dynamics. For species with rapid intrinsic growth rates such as *C. erythraeus* and *P. lotor*, population control measures need to be implemented early to prevent their populations from reaching densities that would result in substantial ecological and economic damage. For slower growing species such as *M. coypus*, the progressive but slower escalation offers a longer intervention window, although delaying action still risks substantial cumulative impacts if populations are left unmanaged.

The contrasting curve types provide complementary insights into the need for timely management to prevent populations from reaching sizes where the economic and ecological impacts become disproportionately large. Our findings refine previous genus‐level assessments (Ahmed, Hudgins, Cuthbert, Haubrock, et al., 2022) that revealed a mixture of cost–density relationships but identified the high‐threshold curve as the most common. By controlling for country‐level differences in cost reporting and socioeconomic activity through a single‐country time series, we show that both high‐threshold and high‐density curves can be relevant at the species level, even within the same taxon (mammals). Although it remains speculative to generalize from only five species, the consistency of these two curve forms as adequate descriptors of cost–density relationships—despite species differing in their ecological traits, range sizes, and impacts—suggests that they are not atypical.

From a management perspective, our findings underline the importance of recognizing which type of cost–density relationship a species follows. High‐threshold species might appear benign at low densities, yet can trigger sudden and severe economic and ecological costs once populations surpass critical sizes. In contrast, high‐density species provide earlier signals of escalating impacts, but their cumulative costs remain substantial if left unmanaged. Distinguishing between these dynamics can help practitioners prioritize preemptive control for high‐threshold invaders, while ensuring sustained containment of high‐density species before costs accumulate to near saturation.

### Interspecific variability and drivers of long‐term economic impacts

Our study highlights the considerable variability in the long‐term economic impacts of non‐native mammals. The large range in maximum cost underscores the need for species‐specific assessments when considering the ecological and economic consequences of non‐native species. These results corroborate the skewed distribution of costs among non‐native species, with global impacts dominated by the negative impacts caused by a few high‐profile taxa (Cuthbert, Diagne, Haubrock, et al., 2022; Jiang et al., 2022; Soto et al., 2022). They further emphasize the complexity of managing biological invasions and provide insights into how ecological characteristics, such as growth rates and environmental adaptability, influence the economic burdens that non‐native species impose over time (Bodey et al., 2025).

The high‐magnitude costs caused by *P. larvata* (US$83.38 million) were likely linked to the species' broad ecological tolerance, its ability to thrive in various environments, and its role in transmitting zoonoses and damaging agricultural systems (Shimoyama & Tsuji, 2024). This is consistent with its high intrinsic growth rate (α = 0.151) and large environmental scaling factor (γ = 1.93), which together indicate both strong reproductive potential and the capacity to exploit resources across environments. Likewise, *P. lotor* (US$57.31 million) is highly adaptable and capable of living in urban environments alongside forests and farms, where it disrupts ecosystems by preying on native species, spreading zoonotic diseases, and competing for resources (Salgado, 2018). Its high γ (2.93) reflects the species' ability to establish from low initial densities, while its high growth rate (α = 0.140) supports rapid expansion once established. Their increasing presence in urban areas has damaged infrastructure, increased waste management costs, and posed potential public health risks (García et al., 2012; Salgado, 2018). Despite the low cost projections for *C. erythraeus* (US$1.17 million), the non‐monetisable ecological impacts of this species, including competition with native squirrel populations and damage to trees, still threaten long‐term forest health, with cascading effects on ecosystem services such as carbon sequestration and biodiversity conservation (Bertolino & Lurz, 2013). Its high growth rate (α = 0.217) suggests that populations can expand rapidly, even though long‐term costs remain low, perhaps reflecting constraints imposed by its smaller range or narrower impact scope. *H. javanicus* has caused declines in native vertebrate populations, leading to changes in food‐web dynamics and reduced ecosystem resilience (Watari et al., 2008). Its low estimated costs could reflect the species' limited distribution in certain Japanese islands and current management success, but the wide confidence intervals suggest that the species could pose a much greater future economic burden if its range expands. However, economic impact estimates for *H*. *javanicus* were based on few cost records, and these wide upper bounds likely reflect uncertainties associated with small sample size. Its low growth rate (α = 0.085) and small γ (1.15) are consistent with both its island‐restricted distribution and lower potential for explosive population growth. In *M. coypus* (US$21.31 million), burrowing activity can have disproportionately large ecological and economic effects as populations increase, with structural damage to levees, riverbanks, and wetlands scaling nonlinearly (Corriale et al., 2006; Dondina et al., 2024). At low densities, burrowing can cause localized erosion, but as population size grows, widespread destabilization of critical infrastructure could occur, leading to costly repairs and habitat degradation. This is consistent with its moderate cost projections, which nevertheless represent a substantial economic burden in affected areas. Its moderate growth rate (α = 0.126) and intermediate γ (2.05) support steady expansion, amplifying damage as densities rise.

Similar dynamics might apply to other ecosystem engineer species, where population expansion intensifies impacts beyond simple numerical increases. For other species we assessed, damages could be affected by density feedback, such as where the viability of agricultural crops or integrity of infrastructure (Suzuki & Ikeda, 2020; Tamura & Yasuda, 2023) can be exacerbated as population sizes pass a threshold that compromises their function. Similarly, for non‐native species implicated in zoonotic infections, risks can increase nonlinearly with initial invader densities owing to the increasing risk of pathogen transmission as densities (and therefore, individual contact rates) increase (Ferraguti et al., 2023).

### Threshold densities

The identification of threshold densities in the damage cost curve has implications for understanding the dynamics of non‐native species' economic impacts based on their population dynamics. The variation in the timing of threshold densities across species underscores the need for tailored management strategies. Although each species exerts pressure on ecosystems in unique ways, the fundamental conclusion is clear: Early intervention is necessary to avoid reaching threshold points, after which costs rise exponentially. The major implications of our model thresholds are that: (1) time lags to cost reporting last decades—even centuries—meaning that initially benign invasions could precipitate substantial economic impacts in the future; (2) there are threshold densities before the rapid escalation of damage costs, where management actions such as rapid eradication should be prioritized to mitigate exponential growth; (3) the rapid cost‐growth phase typically lasts 10–20 years within species at the spatial scales we examined (with *H. javanicus* the only exception, showing a much longer escalation phase of ~35 years, reflecting the limited sample size and fewer cost reports available for this species); and (4) future cost increases can remain substantial even after the point of saturation is reached, notwithstanding the potential for new invasions or impacted sectors to emerge. These thresholds have clear management implications, corroborating the “cost of inaction” that accrues with management delay over time (Ahmed, Hudgins, Cuthbert, Kourantidou, et al., 2022). The variation in the duration of rapid cost escalation reflects the broader ecological and economic implications of species invasions—some species require urgent, short‐term action, while others present more gradual opportunities for intervention. However, the common theme is the need for early detection and management, particularly for those species with rapid, more acute cost escalation. It is also important to consider the feasibility of management interventions at all impact stages, because actions might not be effective in the absence of appropriate techniques or resources (Robertson et al., 2020).

The concept of “near saturation” where damage costs reach 90% of the maximum potential provides another important perspective for management. For species like *M. coypus*, reaching near‐saturation signals that the species' impact on the ecosystem is approaching its peak. This stage represents an ideal point for localized management interventions. Although it might not always be possible to completely reverse the trajectory, management efforts could aim to reduce the population density below the threshold (*z*_thresh_). Ecologists should consider acting at this juncture, even if opportunities were missed at earlier stages, because action can prevent further escalation of costs and reduce the long‐term damage. However, species that reach near saturation more slowly, such as *H. javanicus*, require careful and continuous monitoring to ensure that their impacts do not go unchecked, despite impacts being less acute. Given that some of our results are future projections, it would be unwise to neglect further management, particularly considering the potential for new invasions to reset the dynamic and trigger additional costs. The timeframes associated with threshold and near‐saturation points highlight the importance of establishing early warning systems for non‐native species. These results should further be viewed in the context of the Japanese islands where they were estimated, because invasions into further islands could cause for the high‐threshold cost dynamic to repeat several times, with these “new” subnational invasions not necessarily captured with country‐level first records. The same could be said for economic development, as surges in new sectors could provide novel impact opportunities in non‐native populations.

### Role of management in shaping future damage cost trajectories

Our modeling framework focuses on projecting damage costs over time in a business‐as‐usual scenario. This means that our projections are baseline trajectories, which implicitly assume that management efforts will continue along current trends. However, if management efforts intensify significantly in the future in response to escalating damage, observed costs will likely be lower than our projections, which therefore provide conservative upper bounds for planning purposes.

Complementing previous work on the costs of inaction (Ahmed, Hudgins, Cuthbert, Kourantidou, et al., 2022) and the importance of early management (Bradshaw et al., 2024; Cuthbert, Diagne, Hudgins, et al., 2022; Epanchin‐Niell, 2017; Leung et al., 2002; Lodge et al., 2016), our modeling identifies tipping points beyond which damage costs accelerate sharply. For cost‐effective management, these population thresholds are key indicators beyond which management efforts need to increase to prevent exponential escalation of damages.

Having data on the intensity of management based on observed damage would enable us to refine our projections. It would also enable us to analyze the effects of early management on the temporal dynamics of damage costs. Previous work has shown that delays in management can result in much higher total costs, even when interventions are eventually introduced. For instance, Ahmed, Hudgins, Cuthbert, Kourantidou, et al. (2022) quantified the cost of inaction using a generalized damage–management model and empirical cost data for *Aedes* spp. mosquitoes, revealing that early action could have reduced long‐term losses by billions of dollars. These findings align with a broader literature showing that proactive management is more cost‐effective than delayed or reactive responses (Bradshaw et al., 2024; Cuthbert, Diagne, Hudgins, et al., 2022; Epanchin‐Niell, 2017; Leung et al., 2002; Lodge et al., 2016).

### Model limitations and future research directions

Although our study provides a useful approach to modeling past dynamics and future trajectories of the economic impacts of non‐native species through generalized cost curves, there remain several avenues for improvement.

1. One limitation is data completeness and precision, especially for species such as *H. javanicus* where the scarcity of reported damage costs hampers robust inference. For that species, it was possible to estimate ecologically realistic threshold times for cost escalation, albeit with lower certainty than for the other cases. The long escalation phase we estimate (~35 years) could be much shorter, at least 15 years, with no upper bound. This is likely a product of sparse cost reporting rather than a true reflection of the species' dynamics, emphasizing the need for sufficient data to draw more reliable ecological inferences. Moreover, our study largely relied on data from a single publication, meaning that although standardized, analyses were based on one study's methodology. This scarcity of publications does not necessarily mean that costs were not incurred, but that they were not systematically reported and accessible. Indeed, current reporting of damage costs for many invasions is insufficiently detailed (or entirely absent) for rigorous cost assessments over time. National‐level variation in reporting is also important, with a general need for standardization and expansion of cost reporting to improve model reliability (Diagne et al., 2021; Hulme et al., 2024). Future research over concurrent impact timescales could allow for this variation to be formally captured across multiple publications, thereby helping resolve issues with sample size.
2. Our model currently assumes logistic growth with a fixed carrying capacity, yet ecological systems often display more complex population dynamics, including time‐varying growth rates, fluctuating carrying capacities, and nonlinear behaviors such as boom–bust cycles or chaotic fluctuations (Haubrock et al., 2022; Strayer et al., 2017). Future extensions could incorporate these complexities using stochastic or nonautonomous growth models. In addition, climate and land‐use changes are expected to modify climate suitability, habitat availability, and resource availability, thereby altering parameters like carrying capacity (*K*) and intrinsic growth rate (α) over time. Recent global analyses indicate that climatic suitability for non‐native mammal establishment could shift poleward and expand in many regions, even as natural dispersal potential declines due to mismatched movement capacity and changing conditions (Biancolini et al., 2024). However, species‐specific projections are rare, and no detailed projections exist for our focal mammals in Asia. Future work should therefore pursue targeted modeling that links climate and land‐use scenarios to dynamic parameterization of *K* and α, at species and regional scales, to enable more precise cost and spread projections.
3. Another important omission is the role of management, as our model focuses solely on damage costs and available management data are currently limited and often misaligned with damage data. Consequently, our projections are conditional on historically observed management intensity, even though escalating damage might trigger more intensive interventions, including nonlinear or threshold responses, which could dampen or delay cost accumulation and result in realized future costs that are lower than those projected by a damage‐only model (Ahmed, Hudgins, Cuthbert, Kourantidou, et al., 2022). Using the same filters applied to damage costs, only *P*. *lotor* had reported management costs totaling US$0.07 million between 2014 and 2017, amounting to a negligible 0.60% of the total observed damage cost over the same period. No comparable data were available for the other focal species, although it is likely that variable management is in place. Improved and standardized reporting of management costs would enhance projective capacity by allowing models to account for feedback between control efforts and the ensuing damage–cost dynamics. This is especially relevant when interpreting the steepness or timing of cost accumulation, which could partly reflect underlying control efforts. Including management expenditure data, where available, could help disentangle the extent to which cost curves reflect ecological spread versus suppression effects (Lodge et al., 2006). In the absence of such data, models that incorporate only damage costs, such as the one we present here, provide a useful, albeit conservative estimate of future impacts under scenarios lacking increased management intervention.
4. Another limitation is the assumption of a static maximum cost parameter (*C*_max_). Although the empirical trajectories of our focal mammals support the interpretation of a near‐saturation dynamic, this assumption may not hold in cases where species have not yet fully occupied all suitable areas within the receiving region (Arlé et al., 2024). Future research could explicitly assess whether a species has saturated the suitable conditions using methods such as the cumulative niche framework (Arlé et al., 2024), which evaluates species‐environmental equilibrium. Such analyses could help determine whether the assumption of a static *C*_max_ is appropriate or if long‐term costs are likely underestimated due to the lack of species‐environmental equilibrium.
5. At a broader scale, the damage cost model could be adapted for national‐level assessments, allowing for country‐specific projections that guide local policy and resource allocation, especially in regions with high invasion exposure or economic vulnerability (Zenni et al., 2021). Moreover, there is a strong case for modeling feedback loops between biological invasions and societal responses. For instance, the time it takes for a non‐native species to reach economic thresholds might allow managers to adapt their interventions, thereby altering the trajectory of costs (Ahmed, Hudgins, Cuthbert, Kourantidou, et al., 2022).
6. Developing dynamic threshold models that evolve with intervention intensity and timing would therefore improve both projective realism and policy relevance (Essl et al., 2020; Seebens et al., 2017). Because the magnitude of economic costs can reflect a “lock and key” mechanism between impacted sectors and the invading population's traits (Cuthbert et al., 2025), changes in sectors of economic activity or invader trait changes over time could be further integrated into a dynamic modeling framework to account for modifications in these economic and invasional characteristics. Models could also incorporate trajectories of economic development, because the emergence or intensification of economies might amplify impacts within a given range, even where impacts might otherwise appear to have saturated. Similarly, trait changes in non‐native populations, whether through evolution or environmentally mediated phenotypic plasticity, could reinitiate or exacerbate impacts and require adaptive parameterisation over time (Cuthbert et al., 2025).
7. From a theoretical standpoint, there is also scope to integrate spatiotemporal dynamics using reaction–diffusion models or network‐based approaches. These tools can capture the spread of non‐native populations across heterogeneous landscapes and identify spatial hotspots of risk (Cantrell & Cosner, 2003; Hastings, 1996; Volpert & Petrovskii, 2025). Addressing these directions will enhance our capacity to anticipate, interpret, and manage the economic risks posed by non‐native species in an increasingly interconnected world.

## CONCLUSION

Integrating ecological population dynamics with economic cost modeling can illuminate the latent and nonlinear relationships underlying the future damage costs of biological invasions and the management measures required. By centering the focus on future projections, our modeling framework highlights the economic costs expected to escalate in the coming decades, even for species that appear to have stabilized. Anchoring projections in logistic growth and density feedback‐impact thresholds, the framework reveals an ecological reality: non‐native species often exhibit prolonged periods of minimal detectable impact before costs escalate abruptly. These delayed trajectories, driven by time lags in population establishment and resource exploitation, indicate that although the past dynamics provide important insights, the future escalation of costs remains substantial, challenging conventional risk assessments that prioritize immediate threats over long‐term projections.

This result underscores the importance of monitoring not just population presence or spread, but also the future thresholds at which invasions shift from being benign to destabilizing. The model's reliance on species‐specific growth rates and environmental adaptability highlights the interplay between life‐history traits and invasion outcomes. For example, fast‐growing species require urgent intervention to preempt rapid cost surges, while “slower” invaders might still need long‐term vigilance to avoid future saturation of ecological and economic systems, emphasizing that even species with slow growth rates could drive future costs as they reach their threshold point. These dynamics reinforce the value of mechanistic, trait‐based approaches in invasion ecology, moving beyond static impact assessments to capture the temporal cascades of harm expected in the future.

For policymakers and practitioners, our work emphasizes that early action, guided by projected ecological thresholds, is not just precautionary, but also economically imperative. Even for population‐specific cost trajectories that have reached apparent saturation decades or centuries post‐invasion, we show that the potential for future escalation remains substantial, potentially driven by changes in economic activity, new subnational invasions, or changing population‐level traits. By framing invasions as delayed yet exponential threats, the model compels a shift from reactive to anticipatory governance, particularly in regions with high invasion debt. Ultimately, this synthesis of ecological and economic principles provides a powerful and generalizable framework to confront the escalating biodiversity crisis, urging interdisciplinary collaboration to mitigate the profound, yet often irreversible, costs of inaction and the future impacts of biological invasions.

## AUTHOR CONTRIBUTIONS

Danish A. Ahmed and Corey J. A. Bradshaw conceived the ideas and designed the methodology. Emma J. Hudgins filtered, extracted, and compiled the data from the *InvaCost* database. Danish A. Ahmed and Noor Tahat analyzed the data. Danish A. Ahmed, Corey J. A. Bradshaw, Emma J. Hudgins and Ross N. Cuthbert led the writing of the manuscript. All authors contributed to the ecological interpretation of the findings, and were involved in writing, reviewing, and editing the manuscript.

## CONFLICT OF INTEREST STATEMENT

The authors declare no conflicts of interest.

## Supporting information


Appendix S1.

Appendix S2.

Appendix S3.

Appendix S4.

## Data Availability

Data (Leroy et al., 2022b) are available through the *invacost* R package (v4.1) in Zenodo at https://doi.org/10.5281/zenodo.6653232 and the Comprehensive R Archive Network at cran.r-project.org/package=invacost. The MATLAB code (Ahmed & Bradshaw, 2026) used to generate Figures 2, 3, 4 is available in Zenodo at https://doi.org/10.5281/zenodo.18853628.
