# Supplementary material for: Projecting future damage costs of non‐native species using combined dynamical and cost–density equations
Source: Ecol Appl. 2026 Jul 6;36(5):e70252. doi: 10.1002/eap.70252 (PMC13334257; doi:10.1002/eap.70252)
Supplement: Supplementary file 1 — Appendix S1. [file EAP-36-e70252-s002.pdf]

Appendix S1

Projecting future damage costs of non-native species using combined dynamical and cost-density equations

Danish A. Ahmed, Corey J.A. Bradshaw, Noor Tahat, Emma J. Hudgins, Pierre Courtois, Philip E. Hulme, Yuya Watari, Ali Serhan Tarkan, Ismael Soto, Phillip J. Haubrock, Paride Balzani, Ross N. Cuthbert

Ecological Applications

Abstract in English

Biological invasions threaten biodiversity, economic stability, and public health, exacerbated by intensive global trade and transport. The economic costs of these invasions have exceeded US$ 2 trillion globally and continue to increase. Although past invasion costs have been described across various contexts, there are few robust projections of future costs, limiting effective management planning. We developed a mathematical framework to project future economic damage caused by biological invasions, combining cost-density relationships with a density-time function based on logistic population growth. We tested the model on five well-documented non-native mammal species in Japan, a country with long-term, high-resolution invasion cost records and a well-characterised history of mammal introductions: Pallas' squirrel *Callosciurus erythraeus*, small Indian mongoose *Herpestes javanicus*, nutria *Myocastor coypus*, masked palm civet *Paguma larvata*, and raccoon *Procyon lotor*. Species-level cost–density relationships were characterised by two distinct forms: a high-density curve for *M. coypus* and *P. lotor*, where costs increase progressively with density but the rate of escalation slows at higher densities, and a high-threshold curve for *C. erythraeus*, *H. javanicus*, and *P. larvata*, where costs remain minimal until populations exceed a density threshold, after which they rise steeply. Our model projected accumulated costs to 2050 varying over several orders of magnitude, from $0.43 million (*H. javanicus*) to $88 million (*P. larvata*), with proportional increases ranging from ~ 15% (*M. coypus*) to ~ 78% (*H. javanicus*). Under business-as-usual management, we explicitly model damage-only costs, assuming a historically observed management trend. These projections should therefore be interpreted as maximum estimates. Our approach identifies thresholds beyond which damages escalate rapidly — costs begin to surge 40 to 80 years after the first record, with 90% of expected long-term damages incurred typically within 10 to 20 years. For managers, these results highlight the importance of timely interventions, underscoring the need for tailored management strategies considering species-specific dynamics, socio-economic contexts, and the speed of cost escalation. Early-stage cost dynamics can project future trajectories of existing and emerging invasions, helping guide proactive management prioritisation. Our projections equip policymakers and resource managers with improved foresight to anticipate and mitigate future economic burdens of non-native species across spatial scales and for different taxa.

Abstract in Japanese

外来種の侵入は，生物多様性や経済の安定性，公衆衛生の脅威となり，活発化する国際貿易と輸送によって悪化している．このような，外来種の侵入によりもたらされる経済被害は，世界全体で数兆米ドルに達しており，今後も増加し続けると予想されている．しかし，すでに生じた過去の外来種の侵入による経済被害については，これまでにさまざまな文脈で説明されてきたものの，将来生じうる経済被害に関する信頼性の高い推計はほとんどなく，効果的な管理計画の立案を困難にしていた．本研究では，経済被害と密度の関係と，ロジスティック個体群成長に基づく密度-時間関数とを組み合わせることで，外来種の侵入による将来の経済被害を推計する数理的枠組みを開発した．私たちは外来種の侵入に伴う経済コストに関する長期的かつ高解像度の記録が整備され，哺乳類の導入の歴史もよく解明されている日本の外来哺乳類を対象に，その中で記録の充実している5種の外来哺乳類—クリハラリス *Callosciurus erythraeus*，フイリマングース *Herpestes javanicus*，ヌートリア *Myocastor coypus*，ハクビシン *Paguma larvata*，アライグマ *Procyon lotor*—を用いて，このモデルの検証を行った．種ごとの被害額と密度の関係は，大きくふたつの異なるタイプに分類された．ひとつは，密度の増加に伴って被害額が段階的に上昇するものの，高密度域ではその上昇率が緩やかになる「高密度型カーブ」で，ヌートリアとアライグマが該当した．もう一つは，密度が一定の閾値を超えるまでは被害がほとんど発生せず，閾値を超えると急激に上昇する「高閾値型カーブ」で，タイワンリス，フイリマングース，ハクビシンが該当した．本モデルによる2050年までの累積経済被害の予測値は，数桁規模で変動し，最小で43万ドル（フイリマングース），最大で8800万ドル（ハクビシン）となり，コストの増加率は約15%（ヌートリア）から約78%（マングース）の範囲であった．通常の外来種管理を前提とした本モデルでは，過去に観察された管理の傾向をもとに，外来種の被害に関する被害額のみを明示的にモデル化している．したがって，これらの推計は，最大値として解釈する必要がある．本研究の手法によって，被害が急激に拡大する閾値が明らかになり，最初の外来種の野外での記録から40〜80年後に被害額が急激に増加しはじめ，通常その後10〜20年の間に，想定される長期的被害額の90%が生じることが示された．本研究の結果は，外来種対策の管理者にとって，タイミングを逸しない早期の対応が重要であることを示しており，種ごとの動態や社会経済的な背景，および経済被害の増加スピードを考慮した，きめ細やかな管理戦略の必要性を強調している．侵入初期段階における経済被害額の動態を把握することで，すでに生じている侵入および新たに生じる侵入の将来推移を推計することができ，先手を打った管理の優先順位づけに役立てることができる．本研究の推計は，政策立案者および資源管理担当者に対し，空間スケールや分類群を問わず，外来種による将来的な経済的損害を事前に見積もり，その軽減に備えるための先見性をもたらすものである．
