# Supplementary material for: Projecting future damage costs of non‐native species using combined dynamical and cost–density equations
Source: Ecol Appl. 2026 Jul 6;36(5):e70252. doi: 10.1002/eap.70252 (PMC13334257; doi:10.1002/eap.70252)
Supplement: Supplementary file 2 — Appendix S2. [file EAP-36-e70252-s001.pdf]

Appendix S2

Projecting future damage costs of non-native species using combined dynamical and cost-density equations

Danish A. Ahmed, Corey J.A. Bradshaw, Noor Tahat, Emma J. Hudgins, Pierre Courtois, Philip E. Hulme, Yuya Watari, Ali Serhan Tarkan, Ismael Soto, Phillip J. Haubrock, Paride Balzani, Ross N. Cuthbert

Ecological Applications

Simplification of the damage cost function for high-density and high-threshold cost–density relationships

The general cost–density model (equation 6 in the manuscript) expresses the accumulated damage cost C as a function of the re-scaled population density z, given by:

$$C(z) = aC_{\max}\left(\frac{1}{e^{-10(z-s)}+1} - b\right), \quad \text{for} \quad 0 < z < 1. \tag{S1}$$

On introducing a normalised cost variable $\hat{C} = C/C_{\max}$, this simplifies to:

$$\hat{C}(z) = a\left(\frac{1}{e^{-10(z-s)}+1} - b\right), \quad \text{for} \quad 0 < z < 1, \tag{S2}$$

where

$$a = \frac{1+e^{-c}}{1-b(1+e^{-c})}, \quad b = \frac{1}{1+e^{10s}}, \quad \text{and} \quad c = 10(1-s). \tag{S3}$$

Here, $C_{\max}$ denotes the maximum potential damage cost as the population density reaches the carrying capacity ($z \to 1$). The normalised damage cost $\hat{C}(z)$, expresses costs relative to this maximum, ensuring comparability across species and parameterisations. The parameter s determines the type of cost–density relationship (i.e., low-threshold $s = 0$, low-density $s = 0.25$, sigmoidal $s = 0.5$, high-density $s = 0.75$ and high-threshold $s = 1$), while a and b which are expressed solely in terms of s, govern the shape of the resulting curve.

In the specific case of a **high-density curve** with $s = 0.75$, the parameters reduce to:

$$a = 1 + e^{-2.5}, \quad b = \frac{1}{1+e^{7.5}} \approx 0, \quad \text{and} \quad c = 2.5,$$

and similarly, in the case of a **high-threshold curve**, where $s = 1$, the parameters are:

$$a = 2, \quad b = \frac{1}{1+e^{10}} \approx 0, \quad \text{and} \quad c = 0.$$

Given that for both of these cases $b \approx 0$, equation (S2) simplifies to the following normalised damage cost function:

$$\boxed{\hat{C}(z) = \frac{a}{e^{-10(z-s)}+1}, \quad \text{for } 0 < z < 1.}$$

Here, s and a distinguish between the two cases as described above.
