# Supplementary material for: Projecting future damage costs of non‐native species using combined dynamical and cost–density equations
Source: Ecol Appl. 2026 Jul 6;36(5):e70252. doi: 10.1002/eap.70252 (PMC13334257; doi:10.1002/eap.70252)
Supplement: Supplementary file 3 — Appendix S3. [file EAP-36-e70252-s004.pdf]

Appendix S3

Projecting future damage costs of non-native species using combined dynamical and cost-density equations

Danish A. Ahmed, Corey J.A. Bradshaw, Noor Tahat, Emma J. Hudgins, Pierre Courtois, Philip E. Hulme, Yuya Watari, Ali Serhan Tarkan, Ismael Soto, Phillip J. Haubrock, Paride Balzani, Ross N. Cuthbert

Ecological Applications

Identifying Thresholds

I. *Threshold Point*

The normalised damage cost function for a high-density or high-threshold curve is given by:

$$\hat{C}(z) = a\left(\frac{1}{e^{-10(z-s)}+1}\right), \quad \text{for} \quad 0 < z < 1, \tag{S1}$$

with $a = 1 + e^{-2.5}$, $s = 0.75$ in the case of a high-density curve and $a = 2, s = 1$ in the case of a high-threshold curve. The density at which damage costs begin to escalate rapidly is defined as the point where the rate of change of normalised cost with density exceeds 1. To identify this density, we first compute the derivative of the cost function (equation S1) and set it equal to 1:

$$\frac{d\hat{C}}{dz} = \frac{10ae^{-10(z-s)}}{\left[e^{-10(z-s)}+1\right]^2} = 1. \tag{S2}$$

Letting $w = e^{-10(z-s)}$, equation (S2) can be rearranged to yield a quadratic equation:

$$w^2 + 2(1-5a)w + 1 = 0, \tag{S3}$$

which has the solution:

$$w = 5a - 1 \pm \sqrt{5a(5a-2)}. \tag{S4}$$

Substituting this solution back into the definition of w yields the threshold density:

$$z_{\text{thresh}} = s - \frac{1}{10}\log_e\left(5a - 1 + \sqrt{5a(5a-2)}\right). \tag{S5}$$

For the **high-density curve** ($a = 1 + e^{-2.5},\ s = 0.75$), two mathematical solutions are possible because the rate of change of normalised cost is equal to 1 at both an earlier and a later point along the cost-density curve. We take the positive root, giving $z_{\text{thresh}} \approx 0.534$, as this marks the critical density prior to the onset of rapid cost escalation. For the **high-threshold curve** ($a = 2,\ s = 1$), there is only one solution within the admissible domain ($0 < z < 1$), corresponding to the positive root. In this case the threshold density is $z_{\text{thresh}} \approx 1 - \frac{1}{10}\log_e\left(9 + \sqrt{80}\right) \approx 0.711$.

The corresponding normalised costs at these threshold densities, obtained from equation (S1), are $\hat{C}(z_{\text{thresh}}) \approx 0.111$ for the high-density curve, and $\hat{C}(z_{\text{thresh}}) = 1 - \frac{2}{5}\sqrt{5} \approx 0.106$ for the high-threshold curve. Thus, for the high-density curve, rapid cost escalation begins when the population density reaches about 53.4% of the carrying capacity, by which point approximately 11.1% of the maximum potential damage cost (C_{max}) has already accumulated. In contrast, for the high-threshold curve, costs remain minimal until a critical density of 71.1% of the carrying capacity is reached, at which stage the accumulated cost is 10.6% of C_{max}.

The time at which the population reaches z_{thresh} can be derived from the logistic growth model:

$$z(t) = \frac{1}{1 + (\gamma - 1)e^{-\alpha t}}, \tag{S6}$$

where α is the intrinsic growth rate and γ is the environmental scaling factor.
Rearranging equation (S6) to solve for t when $z = z_{\text{thresh}}$ gives:

$$t_{\text{thresh}} = -\frac{1}{\alpha} \ln \left[\frac{1}{\gamma - 1} \left(\frac{1}{z_{\text{thresh}}} - 1 \right) \right]. \tag{S7}$$

II. *Midpoint and Saturation Point*

To determine the population density z_ε at which the normalised cost reaches a fraction $\varepsilon \in (0, 1)$ of its maximum value, we set $\hat{C} = \varepsilon$ in equation (S1) and rearrange to obtain:

$$z_\varepsilon = s - \frac{1}{10} \log_e \left(\frac{a}{\varepsilon} - 1 \right). \tag{S8}$$

Here, z_ε represents the density corresponding to any specified proportion ε of the potential total damage cost $C_{\max}$. For the high-density curve ($s = 0.75$), the midpoint (half-saturation) occurs at $\varepsilon = 0.5$, giving $z_{\text{mid}} = z_{0.5} \approx 0.735$, while near-saturation occurs at $\varepsilon = 0.9$, giving $z_{\text{sat}} = z_{0.9} \approx 0.910$. For the high-threshold curve ($s = 1$), the corresponding values are $z_{\text{mid}} = z_{0.5} = 1 - \frac{1}{10} \log_e(3) \approx 0.890$ and $z_{\text{sat}} = z_{0.9} = 1 - \frac{1}{10} \log_e\left(\frac{11}{9}\right) \approx 0.980$.

The times at which the population reaches these densities in each case can be obtained from the logistic growth model (equation S6), given as:

$$t_\varepsilon = -\frac{1}{\alpha} \ln \left[\frac{1}{\gamma - 1} \left(\frac{1}{z_\varepsilon} - 1 \right) \right]. \tag{S9}$$

Here, $t_{\text{mid}} = t_{0.5}$ is the time at which the accumulated cost reaches 50% of $C_{\max}$, and $t_{\text{sat}} = t_{0.9}$ is the time at which the accumulated cost reaches 90% of $C_{\max}$.

III. *Inflection Point*

The inflection point is the density at which the acceleration of the normalised damage cost changes sign, marking the transition from an accelerating to a decelerating rate of increase as population density rises. This point is obtained by setting the second derivative of the cost function (equation S1) equal to zero:

$$\frac{d^2\hat{C}}{dz^2} = -\frac{100a\, e^{-10(z-s)} \left(1 - e^{-10(z-s)}\right)}{\left[e^{-10(z-s)} + 1\right]^3} = 0. \tag{S10}$$

Solving for z gives:

$$z_{\text{infl}} = s, \quad \hat{C}(z_{\text{infl}}) = \frac{a}{2}. \tag{S11}$$

For the **high-density curve** ($a = 1 + e^{-2.5}$, $s = 0.75$), the inflection occurs at $z_{\text{infl}} = 0.75$, corresponding to a normalised cost of $\hat{C}(z_{\text{infl}}) \approx 0.541$. This means that when the population reaches 75% of the carrying capacity, approximately 54% of the maximum potential damage cost has already accumulated. Beyond this point, the rate of cost escalation begins to slow as the curve approaches saturation.

The corresponding time at which the population reaches the inflection density can be obtained in the same way as for the threshold point, using equation (S7). Substituting $z_{\text{infl}} = 0.75$ (in place of z_{thresh}) yields

$$t_{\text{infl}} = -\frac{1}{\alpha} \ln \left[\frac{1}{3(\gamma - 1)} \right]. \tag{S12}$$

In the case of the **high-threshold curve** ($a = 2$, $s = 1$), the inflection would occur at $z = 1$. Because the admissible domain is $0 < z < 1$, this point is never reached in practice, and thus no inflection exists within the domain. As a result, the rate of increase in normalised cost remains in the accelerating phase throughout the entire density interval.
