# Supplementary material for: Projecting future damage costs of non‐native species using combined dynamical and cost–density equations
Source: Ecol Appl. 2026 Jul 6;36(5):e70252. doi: 10.1002/eap.70252 (PMC13334257; doi:10.1002/eap.70252)
Supplement: Supplementary file 4 — Appendix S4. [file EAP-36-e70252-s003.pdf]

Appendix S4

Projecting future damage costs of non-native species using combined dynamical and cost-density equations

Danish A. Ahmed, Corey J.A. Bradshaw, Noor Tahat, Emma J. Hudgins, Pierre Courtois, Philip E. Hulme, Yuya Watari, Ali Serhan Tarkan, Ismael Soto, Phillip J. Haubrock, Paride Balzani, Ross N. Cuthbert

Ecological Applications

Table S1. Long-term damage costs and population dynamics.

Number of independent years with recorded damage costs (*n*), cost reporting period, and estimated parameters from model fitting of the temporal damage cost model for five non-native mammal species in Japan. Reported parameters include the long-term accumulated cost (C_{max}, US$ million), environmental scaling factor ($\gamma = K/u_0$, where *K* is the carrying capacity and u_0 the initial population density), intrinsic growth rate (α, year^{-1}), and initial rescaled population density ($z(0) = 1/\gamma$). Model fits are shown for five alternative cost–density relationships: low-threshold curve (s = 0), low-density curve (s = 0.25), sigmoidal curve (s = 0.5), high-density curve (s = 0.75),and high-threshold curve (s = 1). The goodness of fit is quantified by the root mean square error (RMSE; in US$ million), adjusted coefficient of determination (R^2), and Akaike Information Criterion (*AIC*). The best-supported model for each species was identified as the one with the lowest RMSE (in US$ million) and/or lowest AIC, alongside the highest adjusted R^2. Based on this selection criterion, the high-density curve provided the best fit for *M. coypus* and *P. lotor*, while the high-threshold curve provided the best fit for *C. erythraeus*, *H. javanicus* and *P. larvata*. Values in square brackets indicate 95% confidence intervals for the selected model (bolded).

**Species**	*n*	**Cost reporting period**	**Cost-density curve** (shape parameter, *s*)	**Long-term accumulated cost** (US$ million, C_{max})	**Environmental scaling factor** (γ)	**Intrinsic growth rate** (year^{-1}; α)	**Initial re-scaled population density** $z(0) = 1/\gamma$	RMSE	Adjusted R^2	Akaike *(AIC)*
Callosciurus erythraeus	16	2001–2017	Low-threshold (s = 0)	0.98	105.62	0.291	0.010	0.0356	0.9901	-61.65
			Low-density (s = 0.25)	0.99	18.37	0.208	0.054	0.0363	0.9897	-61.06
			Sigmoidal (s = 0.5)	1.05	5.16	0.158	0.194	0.0296	0.9931	-67.51
			High-density (s = 0.75)	1.14	2.70	0.165	0.371	0.0246	0.9953	-73.51
			**High-threshold** (s = 1)	**1.17 [1.08, 1.27]**	**1.98 [1.80, 2.16]**	**0.217 [0.185, 0.249]**	**0.505 [0.463, 0.556]**	**0.0228**	**0.9959**	**-75.93**

**Species**	*n*	**Cost reporting period**	**Cost-density curve** (shape parameter, *s*)	**Long-term accumulated cost** (US$ million, C_{max})	**Environmental scaling factor** (γ)	**Intrinsic growth rate** (year^{-1}; α)	**Initial re-scaled population density** $z(0) = 1/\gamma$	RMSE	Adjusted R^2	Akaike *(AIC)*
Herpestes javanicus	9	2000–2017	Low-threshold ($s = 0$)	0.26	9.66	0.083	0.103	0.0143	0.9095	-51.58
			Low-density ($s = 0.25$)	0.27	3.99	0.058	0.251	0.0143	0.9098	-51.61
			Sigmoidal ($s = 0.5$)	0.28	2.10	0.048	0.475	0.0142	0.9112	-51.75
			High-density ($s = 0.75$)	0.30	1.42	0.057	0.703	0.0140	0.9126	-51.89
			**High-threshold ($s = 1$)**	**0.29 [0.13, 0.46]**	**1.15 [1.07, 1.24]**	**0.085 [≈ 0, 0.201]**	**0.868 [0.810, 0.935]**	**0.0140**	**0.9136**	**-51.99**
Myocastor coypus	18	2000–2017	Low-threshold ($s = 0$)	18.28	45.35	0.222	0.022	0.3708	0.9962	15.09
			Low-density ($s = 0.25$)	18.64	10.32	0.157	0.097	0.3794	0.9960	15.91
			Sigmoidal ($s = 0.5$)	19.75	3.61	0.120	0.277	0.2797	0.9978	4.93
			**High-density ($s = 0.75$)**	**21.31 [20.61, 22.01]**	**2.05 [2.01, 2.09]**	**0.126 [0.118, 0.134]**	**0.488 [0.479, 0.498]**	**0.2250**	**0.9986**	**-2.89**
			High-threshold ($s = 1$)	21.84	1.52	0.168	0.656	0.2251	0.9986	-2.88

**Species**	*n*	**Cost reporting period**	**Cost-density curve** (shape parameter, *s*)	**Long-term accumulated cost** (US$ million, C_{max})	**Environmental scaling factor** (γ)	**Intrinsic growth rate** ($year^{-1}$; α)	**Initial re-scaled population density** $z(0) = 1/\gamma$	RMSE	Adjusted R^2	Akaike *(AIC)*
Paguma larvata	18	2000–2017	Low-threshold ($s = 0$)	58.14	121.71	0.250	0.008	0.9739	0.9974	49.85
			Low-density ($s = 0.25$)	59.30	20.16	0.177	0.050	1.1336	0.9965	55.31
			Sigmoidal ($s = 0.5$)	65.31	5.32	0.128	0.188	0.8297	0.9981	44.08
			High-density ($s = 0.75$)	75.31	2.69	0.124	0.372	0.5630	0.9991	30.12
			**High-threshold** ($s = 1$)	**83.38 [74.48, 88.28]**	**1.93 [1.88, 1.97]**	**0.151 [0.142, 0.161]**	**0.519 [0.508, 0.532]**	**0.5088**	**0.9993**	**26.48**
Procyon lotor	18	2000–2017	Low-threshold ($s = 0$)	45.52	155.64	0.275	0.006	0.8076	0.9974	43.11
			Low-density ($s = 0.25$)	46.33	24.05	0.196	0.042	0.8710	0.9969	45.83
			Sigmoidal ($s = 0.5$)	50.46	5.95	0.143	0.168	0.6407	0.9983	34.77
			**High-density ($s = 0.75$)**	**57.31 [54.40, 60.22]**	**2.93 [2.80, 3.05]**	**0.140 [0.130, 0.150]**	**0.342 [0.327, 0.357]**	**0.5515**	**0.9988**	**29.38**
			High-threshold ($s = 1$)	62.40 [57.50, 67.30]	2.09 [1.98, 2.19]	0.172 [0.156, 0.188]	0.479 [0.456, 0.505]	0.6408	0.9983	34.78
